# Uncovering and Experimental Realization of Multimodal 3D Topological Metamaterials for Low‐Frequency and Multiband Elastic Wave Control

**DOI:** 10.1002/advs.202304793

**Published:** 2023-09-04

**Authors:** Patrick Dorin, Mustafa Khan, K. W. Wang

**Affiliations:** ^1^ Department of Mechanical Engineering University of Michigan Ann Arbor MI 48109 USA

**Keywords:** elastic metamaterial, multiband waveguides, resonance, topological materials, wave control

## Abstract

Topological mechanical metamaterials unlock confined and robust elastic wave control. Recent breakthroughs have precipitated the development of 3D topological metamaterials, which facilitate extraordinary wave manipulation along 2D planar and layer‐dependent waveguides. The 3D topological metamaterials studied thus far are constrained to function in single‐frequency bandwidths that are typically in a high‐frequency regime, and a comprehensive experimental investigation remains elusive. In this paper, these research gaps are addressed and the state of the art is advanced through the synthesis and experimental realization of a 3D topological metamaterial that exploits multimodal local resonance to enable low‐frequency elastic wave control over multiple distinct frequency bands. The proposed metamaterial is geometrically configured to create multimodal local resonators whose frequency characteristics govern the emergence of four unique low‐frequency topological states. Numerical simulations uncover how these topological states can be employed to achieve polarization‐, frequency‐, and layer‐dependent wave manipulation in 3D structures. An experimental study results in the attainment of complete wave fields that illustrate 2D topological waveguides and multi‐polarized wave control in a physical testbed. The outcomes from this work provide insight that will aid future research on 3D topological mechanical metamaterials and reveal the applicability of the proposed metamaterial for wave control applications.

## Graphical abstract

This paper involves the synthesis of a 3D topological metamaterial that harnesses multimodal local resonance to enable multiband and low‐frequency elastic wave control. Theoretical and experimental investigations uncover a methodology to achieve polarization‐, frequency‐, and layer‐dependent waveguides in 3D structures. The reported outcomes provide insight that will encourage future research on 3D mechanical devices for wave and vibration manipulation.

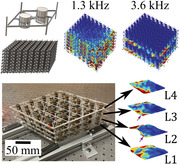

## 1 Introduction

Topological phases have been employed to achieve robust electron transport in quantum systems through conducting states that are protected from local perturbations.^[^ ^1^, ^2^, ^3^, ^4^ ^]^ Recently, topological phases have been integrated into the synthesis of elastic (i.e., mechanical) metamaterials, facilitating extraordinary control over the flow of energy and information contained by elastic waves in mechanical systems. These so‐called topological metamaterials enable low‐loss transport and arbitrary directional manipulation of elastic waves via localized topological states that are protected from unwanted scattering in the presence of structural defects or disorder.^[^ ^5^, ^6^, ^7^, ^8^, ^9^ ^]^ The remarkable capabilities and robustness of topological metamaterials have been exploited to enhance performance in technical applications that include vibration energy harvesters^[^ ^10^, ^11^, ^12^, ^13^, ^14^ ^]^ and a mechanical information processor,^[^ ^15^ ^]^ and have additionally inspired investigations on their potential implementation in on‐chip devices^[^ ^16^, ^17^, ^18^, ^19^ ^]^ and elastic antennas.^[^ ^20^ ^]^

Initial research concerning topological metamaterials focused on the theoretical prediction and experimental demonstration of 0D topological states in 1D mechanical structures (e.g., the wave is localized at a point in a rod) and 1D topological states in 2D mechanical structures (e.g., the wave is localized along a line waveguide in a thin plate).^[^ ^21^, ^22^, ^23^, ^24^, ^25^, ^26^, ^27^, ^28^, ^29^, ^30^, ^31^, ^32^, ^33^ ^]^ Building upon the promising initial outcomes, researchers have begun to explore beyond the traditional 1D and 2D systems to achieve 2D topological states in 3D structures (e.g., the wave is localized along a planar waveguide in a 3D cubic geometry). To construct 3D topological metamaterials, the elastic analogs of Weyl semimetals or the quantum valley Hall effect (QVHE) from electronic systems have been created by carefully configuring the spatial symmetries of 3D periodic lattice geometries.^[^ ^34^, ^35^, ^36^, ^37^, ^38^, ^39^, ^40^ ^]^ The previous research on 3D topological metamaterials has uncovered multiple unprecedented functionalities, including robust elastic wave manipulation along 2D planar waveguides in numerous spatial directions,^[^ ^34^, ^35^, ^36^, ^37^, ^38^, ^39^, ^40^ ^]^ multifaceted wave splitters/networks,^[^ ^34^, ^38^ ^]^ and layer‐selective wave control.^[^ ^34^, ^38^ ^]^

While 3D topological mechanical metamaterials have exhibited the potential to surpass what is possible in 1D and 2D metamaterials, research gaps exist that inhibit their successful implementation in practice. Despite numerous theoretical studies,^[^ ^35^, ^36^, ^37^, ^38^, ^39^, ^40^ ^]^ there is very little experimental evidence of elastic wave control in 3D topological metamaterials,^[^ ^34^ ^]^ due to the challenges associated with the fabrication and testing of intricate 3D mechanical architectures. Furthermore, despite previous studies demonstrating multiband operation in 2D topological metamaterials,^[^ ^41^, ^42^, ^43^, ^44^, ^45^, ^46^ ^]^ the 3D topological metamaterials established thus far are constrained to function in a single frequency band. This single‐band characteristic limits the working bandwidth and information‐carrying capacity of 3D topological metamaterials, reducing their suitability for multiband wave‐based applications such as lasers,^[^ ^47^ ^]^ filters,^[^ ^48^ ^]^ resonators,^[^ ^49^ ^]^ on‐chip circuits,^[^ ^50^ ^]^ isolators,^[^ ^51^, ^52^ ^]^ and wireless networks.^[^ ^53^ ^]^ Finally, the previously developed 3D topological mechanical metamaterials largely function in a high‐frequency regime (i.e., the ultrasonic range: 20 kHz to ≈1 GHz), resulting in a lack of 3D mechanical platforms that accomplish low‐frequency (i.e., few Hz to 20 kHz) topological wave manipulation.

This research addresses the aforementioned gaps and advances the state of the art through the synthesis and experimental realization of a novel 3D topological metamaterial that harnesses multimodal local resonance for low‐frequency and multiband elastic wave control. The geometry of the proposed mechanical metamaterial is configured to obtain four distinct low‐frequency (<6 kHz) topological states derived from multimodal local resonances that can be tailored without changing the lattice constant. Rich polarization‐dependent behavior is encoded into the metamaterial by taking advantage of the multiple unique polarizations of the resonant topological modes. Dispersion analyses and full‐scale response simulations illustrate how these topological states emerge through the QVHE and can be exploited to obtain frequency‐ and layer‐dependent elastic waveguides in 3D structures. Moreover, a comprehensive experimental investigation validates the theoretical predictions and produces the first measurements of complete elastic wave fields and multi‐polarized elastic waveguides in a 3D topological mechanical metamaterial. The findings presented within this paper establish a foundation for further experimental research on 3D topological mechanical metamaterials and illuminate the multifaceted features of the proposed metamaterial that would be valuable for vibration engineering applications.

## 2 Results

### 2.1 3D Metamaterial Description

**Figure 1:**
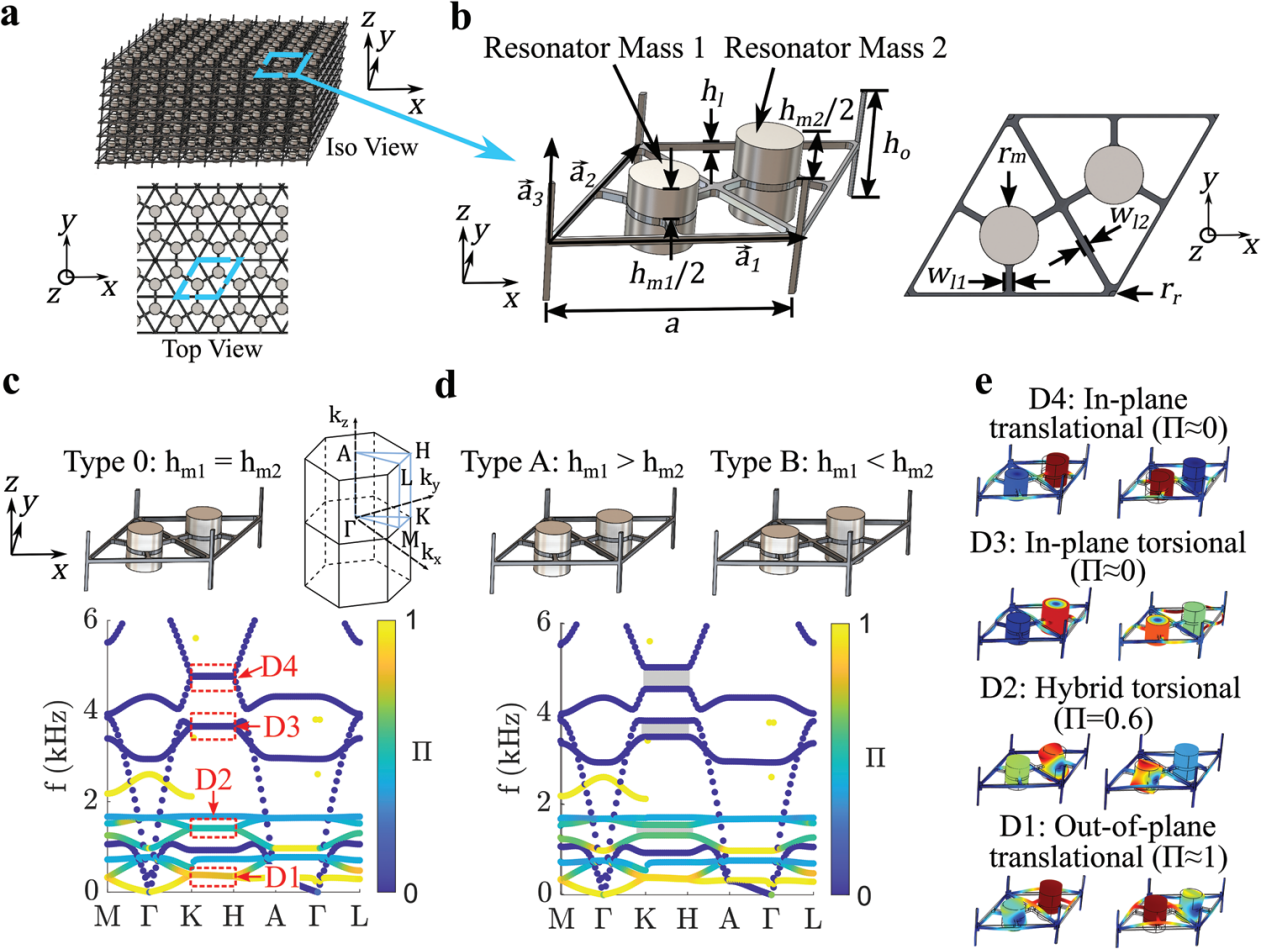
a) A schematic of the 3D topological metamaterial. b) Isometric and top views of the metamaterial unit cell. c) The band structure for the Type 0 lattice (*α* = 0). The four Dirac nodal line degeneracies are indicated by the dotted red boxes. The colorbar indicates the mode polarization quantified by the parameter Π. d) The band structure for the Type A/B (*α* = −0.11/0.11) lattices. The band structures for Type A and Type B lattices are identical and superimposed. The three split Dirac degeneracies are marked by gray shading. e) The mode shapes (taken along K‐H) for the bands that border the four (D1, D2, D3, and D4) topological bandgaps in the |*α*|= 0.11 case, illustrating multimodal resonance.

The proposed metamaterial is a 3D periodic structure with aluminum interconnecting rods (for interconnection in the *z* direction, with a radius *r*_*r*_ = 0.93 mm, *E*_*r*_ = 69 GPa, *ρ*_*r*_ = 2700 kg m^−3^, *ν*_*r*_ = 0.33) and two resonators in the unit cell (**Figure** **1**a,b). The schematic for the unit cell is given in Figure 1b, where *a* = 50 mm is the in‐plane (*x*‐*y*) lattice constant, *h*_*o*_ = 25 mm is the out‐of‐plane (*z*) lattice constant, and ${\vec{a}}_{1}=a\hat{x}$, ${\vec{a}}_{2}=\;a\left(1/2\hat{x}+\sqrt{3}/2\hat{y}\right)$, and ${\vec{a}}_{3}=h_{o}\hat{z}$ are the lattice basis vectors. The resonators are created using cylindrical steel masses (*E*_*m*_ = 200 GPa, *ρ*_*m*_ = 8000 kg m^−3^, *ν*_*m*_ = 0.30) of radius *r*_*m*_ = 7.0 mm and baseline height *h*_*m*_ = 14.3 mm that are attached to aluminum spring ligaments (*E*_*l*_ = 69 GPa, *ρ*_*l*_ = 2700 kg m^−3^, *ν*_*l*_ = 0.33) of height *h*_*l*_ = 1.5 mm and width *w*_*l*1,*l*2_ = 1.5 mm through a circular (*r*_*platform*_ = *r*_*m*_ = 7.0 mm) aluminum platform. Each resonator mass is comprised of two sub‐masses that have heights defined as $h_{m1}=\frac{h_{m}}{2}\left(1-\alpha \right)$ and $h_{m2}=\frac{h_{m}}{2}\left(1+\alpha \right)$, where *α* is the mass height perturbation parameter.

The local resonance phenomenon, which enables wave control near the natural frequencies of the resonators,^[^ ^54^ ^]^ is exploited to obtain multiband and low‐frequency wave dispersion characteristics through the design of the resonant elements. A reticular (i.e., mesh‐like) lattice geometry is created from the spring ligaments and steel is selected as the material for the resonant masses to obtain a large mass ratio $\gamma \;=\frac{m_{m}}{m_{l}+m_{r}}$ within a reasonable volumetric envelope, where *m*_*m*_ is the cumulative mass of the steel masses, *m*_*l*_ is the cumulative mass of the aluminum ligaments, and *m*_*r*_ is the cumulative mass of the aluminum interconnecting rods. A mass ratio value that is greater than unity (*γ* = 13.9 with the baseline design parameters) is deliberately attained to ensure that the resonant masses provide a significant impact on the dynamic response of the proposed metamaterial. Previous works have shown that the magnitude of the mass ratio, which governs the relative influence of the resonant elements on the system response, plays a crucial role in achieving broad low‐frequency bandgaps^[^ ^54^, ^55^ ^]^ and effective topological wave propagation^[^ ^56^, ^57^ ^]^ in locally resonant mechanical metamaterials. Furthermore, previous research has revealed that multimodal resonances can be harnessed to create multiple low‐frequency bandgaps in mechanical metamaterials.^[^ ^52^, ^58^, ^59^ ^]^ In the proposed 3D topological metamaterial, the large mass ratio and relative compliance of the mesh‐like aluminum lattice structure facilitate the achievement of multiple low‐frequency out‐of‐plane and in‐plane translational and torsional resonances. This rich set of resonances opens opportunities for multi‐polarized and multiband responses in a low‐frequency regime.

The interconnecting rods create a structural coupling between the 2D layers that contain the mass‐spring resonators (Figure 1a,b), providing a mechanism for elastic waves to propagate along the *z* direction in the 3D structure. The geometry and material properties of the interconnecting rods may be used to modify the wave propagation characteristics of the metamaterial. The metamaterial lattice is arranged into a honeycomb pattern in the *x*‐*y* plane such that it contains *D*_*6* *h*_lattice symmetry (see the top view in the lower panel of Figure 1a), which is used to acquire nontrivial topological features through the QVHE.^[^ ^60^, ^61^, ^62^, ^63^ ^]^ A detailed numerical analysis of the design considerations outlined in this section is provided in Section 2.2.

### 2.2 Unit Cell Dispersion and Multimodal Resonance Effect

The wave dispersion characteristics of the unit cell are analyzed first to investigate the topological characteristics and unique wave propagation features of the proposed metamaterial. As shown in Figure 1c, a unit cell with equivalent mass heights (*α* = 0) is designated as a Type 0 lattice. The band structure is calculated using the commercial finite element solver COMSOL Multiphysics (see Section S1, Supporting Information, for further details on the simulation methods). The *D*_6*h*_symmetry present in the Type 0 lattice leads to four distinct Dirac nodal line degeneracies appearing along the K‐H line in the band structure across a wide frequency range (Figure 1c).^[^ ^1^, ^6^, ^62^ ^]^ These Dirac degeneracies are labeled as D1, D2, D3, and D4 in order of lowest to highest frequency, *f*_*d*−*D*1_ = 0.37 kHz, *f*_*d*−*D*2_ = 1.41 kHz, *f*_*d*−*D*3_ = 3.66 kHz, and *f*_*d* −*D*4_ = 4.76 kHz, which is computed at the midpoint of the K‐H line. A polarization parameter $\Pi \;=\frac{\;\;{\;\int \int \int \;}_{V_{U}}|w{|}^{2}\mathrm{dV}}{\;{\;\int \int \int \;}_{V_{U}}|u{|}^{2}+\left|v{|}^{2}+\right|w{|}^{2}\mathrm{dV}}$  is defined and measured for each mode in the band structure, where *V*_*U*_ is the volume of the unit cell, and *u*, *v*, and *w* are the displacement components in the *x*, *y*, and *z* directions, respectively. The Dirac degeneracies in Figure 1c each contain different polarizations, with D1 being predominantly out‐of‐plane polarized (Π ≈ 1, yellow in Figure 1c), D2 having a hybrid polarization (Π = 0.5, green in Figure 1c), and D3/D4 containing in‐plane polarized displacements (Π ≈ 0, blue in Figure 1c). To follow the requirements of the QVHE,^[^ ^60^, ^61^, ^62^, ^63^ ^]^ the *D*_6*h*_ symmetry is reduced to *D*_3*h*_ symmetry by perturbing the mass heights from the baseline configuration (*α* ≠ 0). The lattice is designated as Type A for *α* < 0 or Type B for *α* > 0 (Figure 1d). Calculation of the band structure for the cases of *α* = ±0.11 reveals that this reduction in symmetry splits the Dirac degeneracies and leads to bandgaps opening along the K‐H line (Figure 1d, note that the Type A and Type B band structures are superimposed, as they are identical for |*α*| = 0.11). For the case of |*α*| = 0.11, partial bandgaps are obtained from the Dirac degeneracies D2, D3, and D4 that cover the frequency ranges of *f* = 1.3–1.5 kHz, *f* = 3.5–3.8 kHz, and *f* = 4.5–5.1 kHz, respectively. The split D2, D3, and D4 degeneracies and the frequency ranges of the partial bandgaps that are opened from them are represented with gray shading in Figure 1d. A partial bandgap may also be opened from D1 if the mass perturbation |*α*| is increased further (see the evolution of all four bandgaps with respect to |*α*| and a D1 topological state for |*α*| = 0.40 in Section S2, Supporting Information). While the bandgaps are incomplete (i.e., they do not cover the entire Brillouin zone of reciprocal space), previous works have demonstrated that complete bandgaps are not strictly required for effective topological wave control using the QVHE,^[^ ^64^, ^65^, ^66^ ^]^ so long as there is no coupling between the non‐topological bands that cross through the partial bandgaps and the topological bands of interest. Further discussion clarifying why the partial nature of the bandgaps does not inhibit the construction of topological waveguides in the proposed metamaterial is included in later sections.

The topological nature of the D2, D3, and D4 bandgaps is evaluated by computing the valley Chern number $C_{v-p}=\frac{1}{2\pi }{\;\int \int \;}_{v}B_{p}\left(k\right)d^{2}k$ for the bands bordering each bandgap, where *B*_*p*_(*k*) is the Berry curvature, *p* = 1 refers to the band delineating the low‐frequency bandgap boundary, and *p* = 2 refers to the band delineating the high‐frequency bandgap boundary (see Section S3, Supporting Information for more details on the *C*_*v*−*p*_ calculations). The resulting *C*_*v*−*p*_ are $C_{v-1}^{\mathrm{Type}\;A}$ = 0.11, $C_{v-2}^{\mathrm{Type}\;A}$ = −0.09, $C_{v-1}^{\mathrm{Type}\;B}$ = −0.11, $C_{v-2}^{\mathrm{Type}\;B}$ = 0.09 for the D2 bandgap; $C_{v-1}^{\mathrm{Type}\;A}$ = −0.14, $C_{v-2}^{\mathrm{Type}\;A}$ = 0.17, $C_{v-1}^{\mathrm{Type}\;B}$ = 0.15, $C_{v-2}^{\mathrm{Type}\;B}$ = −0.18 for the D3 bandgap; and $C_{v-1}^{\mathrm{Type}\;A}$ = 0.25, $C_{v-2}^{\mathrm{Type}\;A}$ = −0.30, $C_{v-1}^{\mathrm{Type}\;B}$ = −0.26, $C_{v-2}^{\mathrm{Type}\;B}$ = 0.30 for the D4 bandgap. The nonzero *C*_*v*−*p*_ values reveal the topological characteristic for each bandgap, while the equal and inverted *C*_*v*−*p*_ calculated for the Type A and Type B configurations indicate that they are topologically distinct. These nontrivial *C*_*v*−*p*_ magnitudes confirm that topological protection is present in the metamaterial. However, they are less than the idealized |*C*_*v*−*p*_| = 0.5 due to the large amplitude of the perturbation to the mass height.^[^ ^31^, ^66^, ^67^, ^68^ ^]^ Previous research has shown that a 0 <|*C*_*v*−*p*_| < 0.5 does endow a sufficient level of topological protection for wave propagation and indicates that the amplitude of the mass perturbation |*α*| could be reduced to increase |*C*_*v*−*p*_| if necessary.^[^ ^31^, ^66^, ^67^, ^68^ ^]^ While calculations shown in Section S2 (Supporting Information) demonstrate that the bandgaps could be further broadened by increasing |*α*|, |*α*| = 0.11 is selected for the presented results to conserve the calculated |*C*_*v*−*p*_| values. In practice, the tradeoff between bandgap width and |*C*_*v*−*p*_| must be considered when specifying |*α*|.

Further analysis of the unit cell dispersion uncovers the multimodal resonant characteristic of the proposed 3D metamaterial and elucidates how this feature enables low‐frequency topological bandgaps over multiple frequency bands. The mode shapes for the bands bordering each of the four topological bandgaps in the |*α*|= 0.11 case are shown in Figure 1e. All of these mode shapes contain displacement that is largely confined to the resonator masses, a distinguishing characteristic of the local resonance mechanism for bandgap formation.^[^ ^54^, ^69^ ^]^ Furthermore, each set of modes displays a distinct resonant behavior: out‐of‐plane translational for D1 (Π ≈ 1), hybrid torsional for D2 (Π = 0.6), in‐plane torsional for D3 (Π ≈ 0), and in‐plane translational for D4 (Π ≈ 0), illustrating how the engineered multimodal resonance of the proposed metamaterial is the source of the four distinct topological bandgaps (see Section S4, Supporting Information, for further details on these modes).

**Figure 2:**
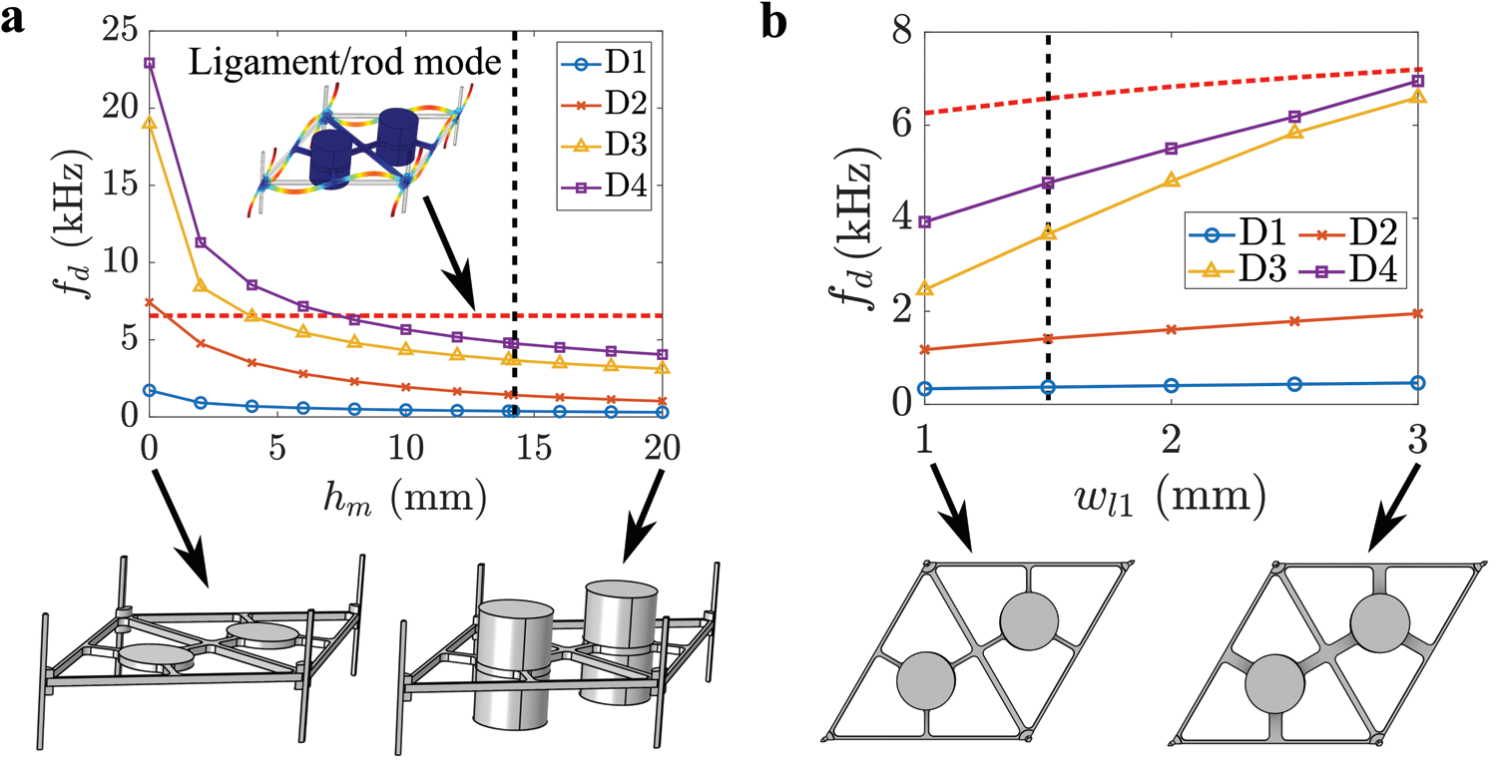
Parameter study illustrating the effect of the a) mass height *h*_*m*_ and b) spring ligament width *w*_*l*1_ on the Dirac nodal line frequency *f*_*d*_, which is taken at the midpoint between K and H for each D1‐D4 (*f*_*d−D1*_, *f*_*d−D2*_, *f*_*d−D3*_, and *f*_*d−D4*_). All presented values are for the Type 0 lattice configuration (*α* = 0). The insets show the unit cell geometries for the minimum and maximum specified values of *h*_*m*_ and *w*_*l*1_. The vertical dashed black lines indicate the specified *h*_*m*_ and *w*_*l*1_ for all presented results in this paper. The dashed red lines indicate the minimum frequency of the ligament/rod modes.

A parameter study is conducted to study the influence of the multimodal resonator design on the Dirac degeneracies D1‐D4 presented in Figure 1c for the *α* = 0 case. Results from this parameter study (shown in **Figure** **2**) illustrate how the respective frequencies *f*_*d*−*D*1_, *f*_*d*−*D*2_, *f*_*d*−*D*3_, and *f*_*d*−*D*4_ of the four Dirac degeneracies can be tailored by adjusting the frequency characteristics of the resonators. Figure 2a reveals that the Dirac frequencies have an inverse relationship with the resonator mass, which is adjusted using the mass height parameter *h*_*m*_. By increasing the resonator mass height, the Dirac frequencies for D1‐D4 can be dramatically reduced, and the four degeneracies can be spectrally separated from extraneous high‐frequency modes that would make it difficult to construct topological bandgaps (and furthermore, effective topological waveguides within those bandgaps). The lowest frequency of the extraneous modes is 6.5 kHz for all *h*_*m*_ (i.e., it is not affected by *h*_*m*_) and is marked by the horizontal dashed red line in Figure 2a. These modes are dominated by displacements of the outer aluminum ligaments (with width *w*_*l*2_ in Figure 1b) and interconnecting rods that contain a hybrid polarization (Π≈0.65, see an example mode in the inset of Figure 2a), and are thus referred to as ligament/rod modes. For the given system parameters, a minimum mass height of *h*_*m*_ = 8.0 mm (corresponding to a mass ratio of *γ* = 7.8) is required to ensure that all four *f*_*d*_ are below the 6.5 kHz threshold. The mass height of *h*_*m*_ = 14.3 mm (marked by the vertical dashed black line in Figure 2a, corresponding to *γ* = 13.9) is selected in this study to ensure that the Dirac degeneracies, and thus the topological bandgaps that are opened from them, are well isolated from the ligament/rod modes. Further details on how interaction with the ligament/rod modes can inhibit the formation of topological bandgaps/waveguides and how these undesirable modes can be separated from the Dirac degeneracies are given in Section S5 (Supporting Information). By employing the local resonance mechanism to isolate the Dirac dispersions in this way, the proposed metamaterial unclutters the intrinsically dense band structure of 3D elastic metamaterials, which has often impeded low‐frequency and multiband topological wave control in previous investigations.

In contrast to the inverse relationship with the resonant mass, the Dirac frequencies exhibit a direct relationship to the stiffness of the resonator spring, which is modified through the ligament width *w*_*l*1_ (Figure 2b). Notably, the *f*_*d*_ for D3 (*f*_*d* − *D*3_) is the most affected by increasing *w*_*l*1_ from 1 to 3 mm (168% increase in *f*_*d*−*D*3_, compared to 37% for *f*_*d*−*D*1_, 66% for *f*_*d*−*D*2_, and 77% for *f*_*d* − *D*4_). This large shift in *f*_*d*−*D*3_ is due to the heightened sensitivity of the in‐plane torsional mode to *w*_*l*1_, compared to the other three sets of modes presented in Figure 1e. The cause of this greater sensitivity can be clarified by treating the spring ligaments as idealized cantilevered beams of length *L*_*l*_. Under this assumption, the bending stiffness for the D1 mode is $S_{D1}=\frac{3w_{l1}h_{l}^{3}}{4L_{l}^{3}}$,^[^ ^70^ ^]^ while for D3 $S_{D3}=\frac{3h_{l}w_{l1}^{3}}{4L_{l}^{3}}$,^[^ ^70^ ^]^ explaining the dramatically enhanced influence of *w*_*l*1_ on the frequency *f*_*d*−*D*3_ of the D3 degeneracy when compared with *f*_*d*−*D*1_. These findings show how the unique stiffness mechanisms for each of the four resonant modes in the multimodal design can be exploited to tune the Dirac frequencies (and thus topological bandgap frequencies) for D1, D2, D3, and D4 relative to each other. In selecting a proper *w*_*l*1_, care must be taken to ensure that all four Dirac degeneracies remain below the dashed red line in Figure 2b, which indicates the lower frequency boundary of the ligament/rod modes. For the *w*_*l*1_ = 1.5 mm chosen in this study (indicated by the vertical dashed black line in Figure 2b), the Dirac degeneracies *f*_*d* − D1_, *f*_*d*−*D*2_, *f*_*d*−*D*3_, and *f*_*d*−*D*4_ are well removed from the ligament/rod modes. Apart from establishing the design considerations of the proposed metamaterial, the results of the presented unit cell parameter studies illuminate how the multimodal resonance of the 3D metamaterial enables topological bandgaps at low frequencies that can be controlled without needing to alter the lattice constants *a* or *h*_*o*_, a significant advantage for volume‐constrained applications.

### 2.3 Supercell Analysis for 2D Topological States

**Figure 3:**
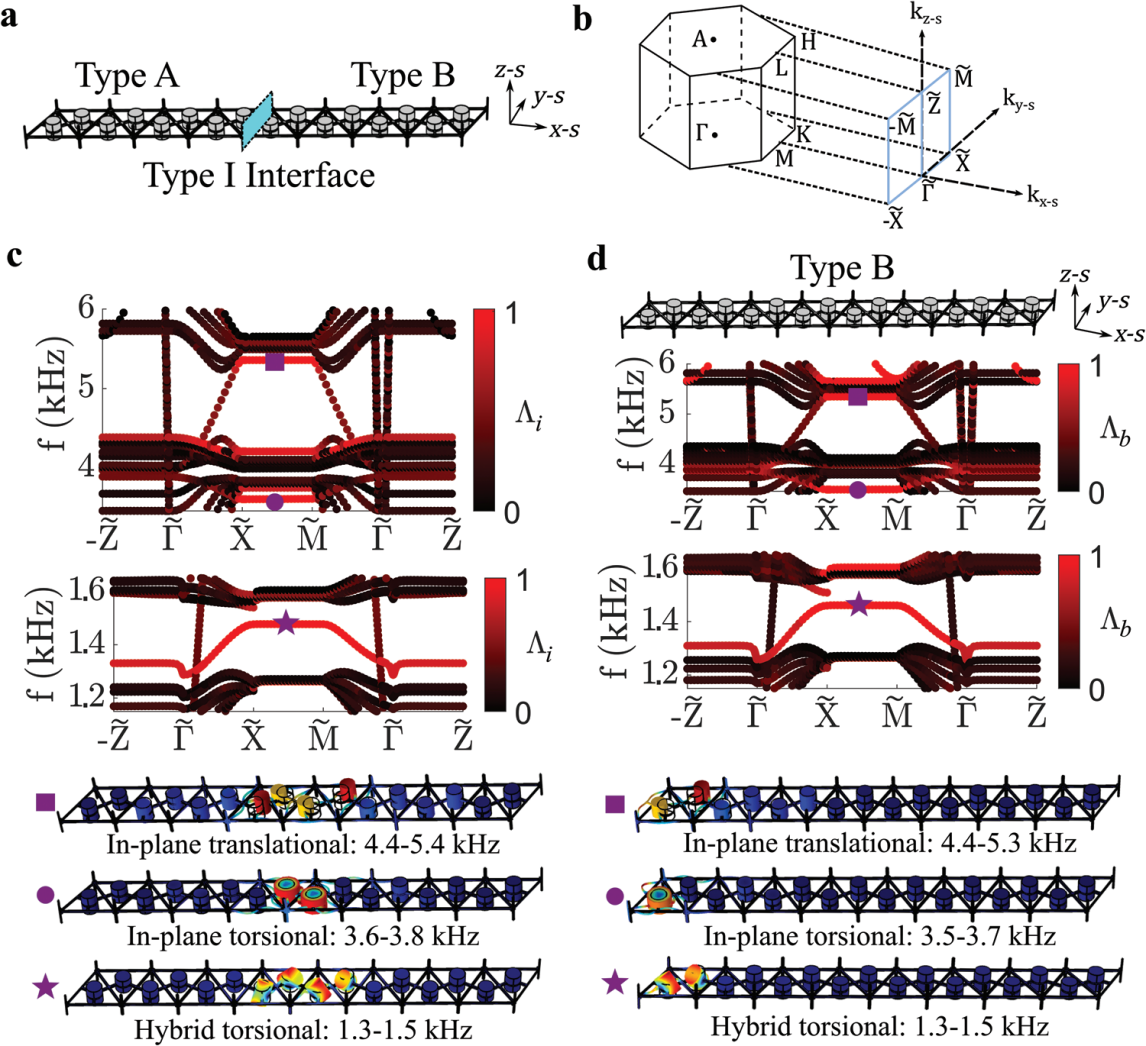
a) Schematic of an eight‐unit supercell with a Type I interface indicated by the blue planar surface. b) The reciprocal space, with one‐half of the surface Brillouin zone projected onto the *k*_*y*−*s*_ − *k*_*z*−*s*_plane outlined in light blue. c) The band diagram for the supercell presented in (a). The red bands (Λ_*i*_ ≈ 1) are interface modes and the black bands (Λ_*i*_ ≈ 0) are bulk modes. Representative mode shapes for the hybrid torsional (purple star), in‐plane torsional (purple circle), and in‐plane translational (purple square) topological interface states are shown at the bottom. d) The schematic and band diagram for a supercell comprised of eight Type B unit cells. The red bands (Λ_*b*_ ≈ 1) are boundary modes and the black bands (Λ_*b*_ ≈ 0) are bulk modes. Representative mode shapes for the topological boundary states are shown at the bottom.

To obtain 2D topological states, an eight‐unit supercell is constructed that consists of four Type A unit cells connected to four Type B unit cells at a Type I interface (**Figure** **3**a). Floquet periodic boundary conditions are applied along the *y*−*s* and *z*−*s* directions, while the left and right boundaries are left free. According to the bulk‐boundary correspondence,^[^ ^1^, ^6^ ^]^ 2D topological states with displacement localized at the interface (i.e., interface states) are expected to emerge within the topological bandgaps, since the Type A and Type B lattices are topologically distinct. The band structure for the supercell is calculated along the surface Brillouin zone projected onto the *k*_*y*−*s*_ − *k*_*z*−*s*_ plane (Figure 3b,c). A localization parameter $\Lambda_{i}={\;\int \int \int \;}_{V_{\mathrm{inte}\mathrm{rface}}}\;d^{2}\;\mathrm{dV}/\;{\;\int \int \int \;}_{V_{S}}d^{2}\mathrm{dV}$  (where *V*_*interface*_ is the volume of the two unit cells at the interface and *V*_*S*_ is the total volume of the supercell) is defined to measure the confinement of the total displacement $d\;=\sqrt{|u{|}^{2}+\left|v{|}^{2}+\right|w{|}^{2}}\;$at the interface for each eigenmode. Thus, interface modes have Λ_*i*_ ≈ 1 (represented by the red bands in the band structure) and bulk modes have Λ_*i*_ ≈ 0 (represented by the black bands in the band structure). As shown in Figure 3c, topological interface states emerge within the D2, D3, and D4 partial bandgaps, satisfying the bulk‐boundary correspondence. Representative mode shapes for each topological interface state are shown at the bottom of Figure 3c, illustrating hybrid torsional (found over a frequency range of 1.3–1.5 kHz), in‐plane torsional (3.6–3.8 kHz), and in‐plane translational (4.4–5.4 kHz) modes with displacement fields that match the unit cell resonant modes for D2, D3, and D4 displayed in Figure 1e. Similarly, a supercell is constructed from eight Type B unit cells, and the resulting band structure is shown in Figure 3d. The band structure for the Type B supercell is nearly identical to that of the supercell with the Type I interface. A localization parameter $\Lambda_{b}={\;\int \int \int \;}_{V_{\mathrm{boun}\mathrm{dary}}}\;d^{2}\;\mathrm{dV}/\;{\;\int \int \int \;}_{V_{S}}d^{2}\mathrm{dV}$ (where *V*_*boundary*_ is the volume of the unit cells at the two supercell boundaries) is defined to measure the modal displacement contained at the boundaries of the Type B supercell. Hybrid torsional, in‐plane torsional, and in‐plane translational topological states are uncovered with displacements that are trapped at the left boundary of the supercell (Λ_*b*_ ≈ 1, Figure 3d). Similar observations of topological boundary states that emerge due to a topological transition at the boundary of a lattice are reported in several previous works and predicted by the bulk‐boundary correspondence.^[^ ^1^, ^6^, ^22^, ^65^, ^66^, ^71^, ^72^ ^]^ These topological boundary states emerge within the D2, D3, and D4 topological bandgaps and align with the frequency ranges for the interface states reported in Figure 3c. The findings gleaned from this supercell analysis reveal that the multimodal resonance of the proposed metamaterial facilitates the achievement of multiband topological interface and surface states.

Further inspection of the band structures in Figure 3c,d reveals that there are bulk modes that cross through the gray D2, D3, and D4 partial bandgaps. While care must be taken to excite the D3 topological interface state without activating the coexisting bulk modes (e.g., by specifically exciting the structure at the location of maximum displacement amplitude for the D3 topological state, see Section 2.4), the bulk modes that cross through the D2 and D4 partial bandgaps near $\tilde{\Gamma }$ contain polarizations that preclude unwanted interactions with the topological states (see more details in Section S6, Supporting Information). Another important feature of the supercell band structure is the sharp notch (with a steep slope) that is observed in the hybrid torsional (1.3–1.5 kHz) topological states for the $-\tilde{Z}-\tilde{\Gamma }$ and $\tilde{\Gamma }-\tilde{Z}$ directions. Since this steep slope (i.e., large group velocity) occurs near $\tilde{\Gamma }$ and the band is flat for the rest of the $-\tilde{Z}-\tilde{\Gamma }-\tilde{Z}$ wavenumber region, this indicates that the interconnecting rods undergo quasi‐rigid body (or very long wavelength) motion that transmits wave energy along the *z* direction (see Figure S9, Supporting Information). In contrast, the two in‐plane topological states are flat (i.e., zero group velocity) and/or gapped for the entire $-\tilde{Z}-\tilde{\Gamma }-\tilde{Z}$ wavenumber region, and thus do not transmit wave energy along *z*. As is shown and discussed in Section S6 (Supporting Information), this dichotomy in *z* direction transport behavior occurs because the hybrid torsional modes couple with the motion of the interconnecting rods, while the in‐plane torsional and in‐plane translational resonant modes do not. This distinction in *z* direction response for the three different topological states opens the door to polarization‐ and layer‐dependent wave control functionality in full‐scale 3D structures.

### 2.4 Topological Waveguides in Full‐Scale 3D Metastructures

**Figure 4:**
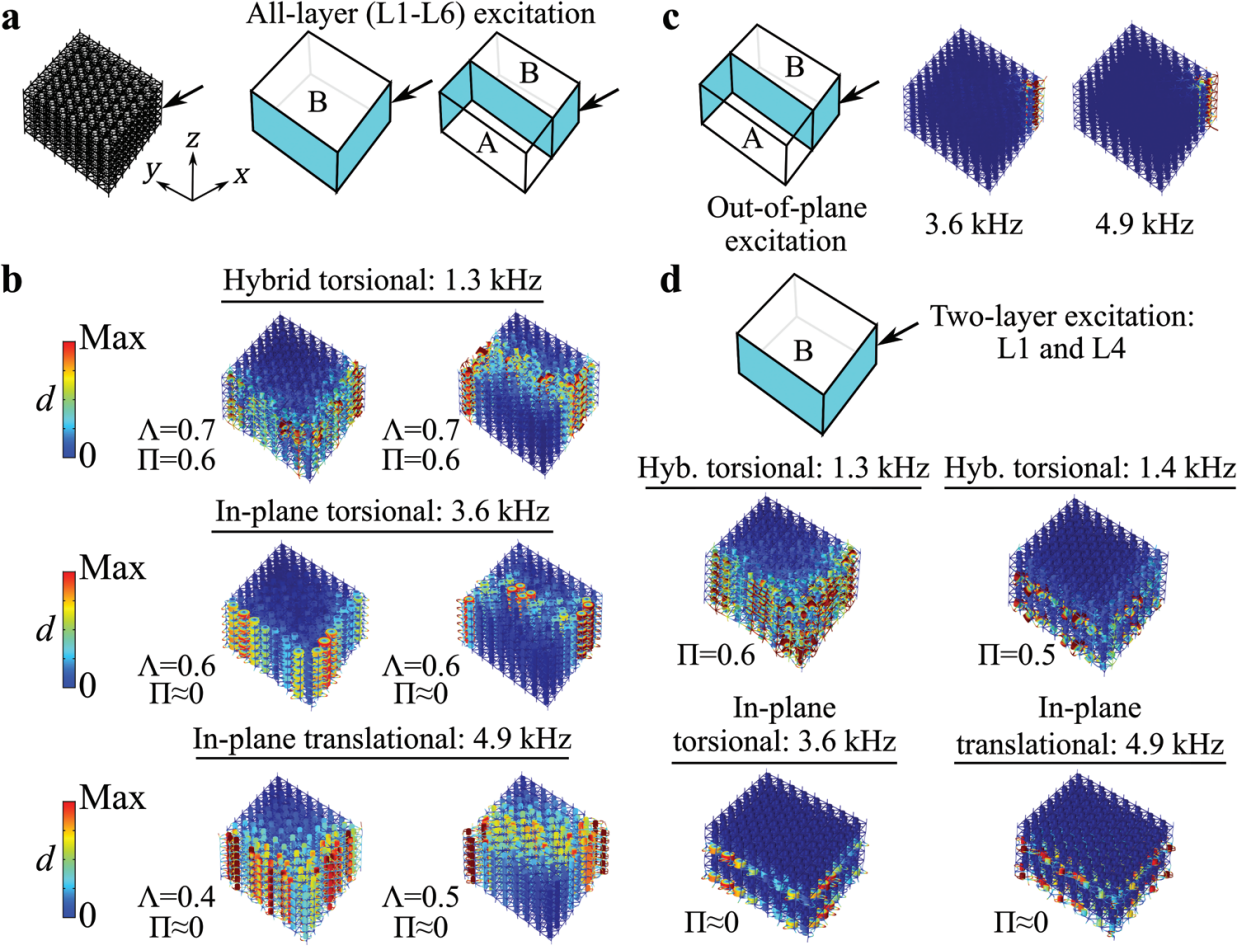
a) Schematic of a full‐scale 3D metastructure constructed from an 8 × 8 × 6 pattern of the metamaterial unit cell (left) and illustrations of V‐shaped (middle) and Z‐shaped (right) waveguides. The distribution of Type A and Type B unit cells is denoted by the letters “A” and “B.” b) Steady‐state displacement fields illustrating waveguides for all‐layer (L1‐L6) input excitations of 1.3 kHz (hybrid torsional), 3.6 kHz (in‐plane torsional), and 4.9 kHz (in‐plane translational). c) Wave attenuation when an out‐of‐plane excitation is used in the frequency ranges of the in‐plane topological states. d) Steady‐state displacement fields illustrating layer‐locked waveguiding for two‐layer (L1 and L4) input excitations of 1.4 kHz (hybrid torsional), 3.6 kHz (in‐plane torsional), and 4.9 kHz (in‐plane translational). For a two‐layer input of 1.3 kHz, the hybrid torsional state exhibits wave transmission across all six layers.

Full‐scale finite element simulations are conducted to investigate the elastic wave control capabilities of the metamaterial in finite 3D metastructures (a metastructure is defined in this paper as a mechanical structure with finite boundary conditions that is created from the metamaterial). A 3D metastructure is constructed from an 8 × 8 × 6 tessellation of the metamaterial unit cell (**Figure** **4**a). To identify each layer in the 3D metastructure, the naming convention of “LX” for layer “X” is used, where layer 1 (L1) refers to the bottom layer in the *z* direction. The four corners at the base of the metastructure are fixed and all other boundaries are left free. Two different elastic waveguides are created in the metastructure by specifying the distribution of Type A and Type B unit cells. The V‐shaped waveguide (blue shading in the middle schematic in Figure 4a) is created from the topological boundary states, while the Z‐shaped waveguide (blue shading in the right schematic in Figure 4a) is generated by connecting the topological boundary and interface states in series. A harmonic excitation is placed on all six metastructure layers (L1‐L6) at one end of the waveguide. The excitation polarization and frequency are selected to activate each of the three unique topological states: out‐of‐plane at 1.3 kHz for the hybrid torsional state, in‐plane at 3.6 kHz for the in‐plane torsional state, and in‐plane at 4.9 kHz for the in‐plane translational state. The resulting steady‐state displacement fields (Figure 4b) reveal a dynamic vibration response that follows the designated 2D waveguides and is polarized according to the corresponding topological state (Π = 0.6 for the hybrid torsional waveguides and Π ≈ 0 for the in‐plane waveguides). The degree of waveguide localization, as quantified by $\Lambda ={\;\int \int \int \;}_{V_{\mathrm{wave}\mathrm{guide}}}\;d\;\mathrm{dV}/\;{\;\int \int \int \;}_{V_{\mathrm{all}}}d\mathrm{dV}$  (where *V*_*waveguide*_ is the volume of the waveguide region, as defined by the unit cells adjacent to the specified waveguide route, and *V*_*all*_ is the volume of the entire 3D metastructure) varies for the three different topological states. At the given frequencies, the hybrid torsional waveguides both have Λ = 0.7, the in‐plane torsional waveguides both have Λ = 0.6, and the in‐plane translational waveguides have Λ = 0.4 and Λ = 0.5. These topological waveguides exhibit unconventional polarization‐ and frequency‐dependent behaviors. For example, if an out‐of‐plane excitation is applied in the frequency range for an in‐plane topological state, the wave is attenuated by the topological bandgap and will not propagate beyond the input location (Figure 4c). Moreover, if the input excitation is provided on layers 1 and 4 (L1 and L4) of the metastructure, the in‐plane topological states display layer‐locked wave propagation (Figure 4d). The dynamic response is locked to L1 and L4 and there is no transfer of wave energy along the *z* direction, which aligns with the supercell band structure for these two topological states. In the case of the hybrid torsional state, the waves propagate along all six layers for a two‐layer input with a frequency of 1.3 kHz and undergo layer‐locked transport if the two‐layer input frequency is increased to 1.4 kHz (Figure 4d). This frequency‐selective behavior is explained by examining the supercell band structure, which predicts that the waves will propagate in the *z* direction for 1.28–1.33 kHz and exhibit more layer‐locked behavior from 1.33 to 1.46 kHz due to the shape of the band over $-\tilde{Z}-\tilde{\Gamma }-\tilde{Z}$ (Figure 3c,d, see more detail in Section S6, Supporting Information). These results illuminate how the multimodal topological states of the 3D metamaterial can be harnessed for polarization‐, frequency‐, and layer‐dependent wave control in finite 3D metastructures. As shown in Section S7 (Supporting Information), this multimodal mechanism can also be exploited in a 2D topological metamaterial, underscoring the generalizability of the proposed concept.

### 2.5 Experimental Realization

**Figure 5:**
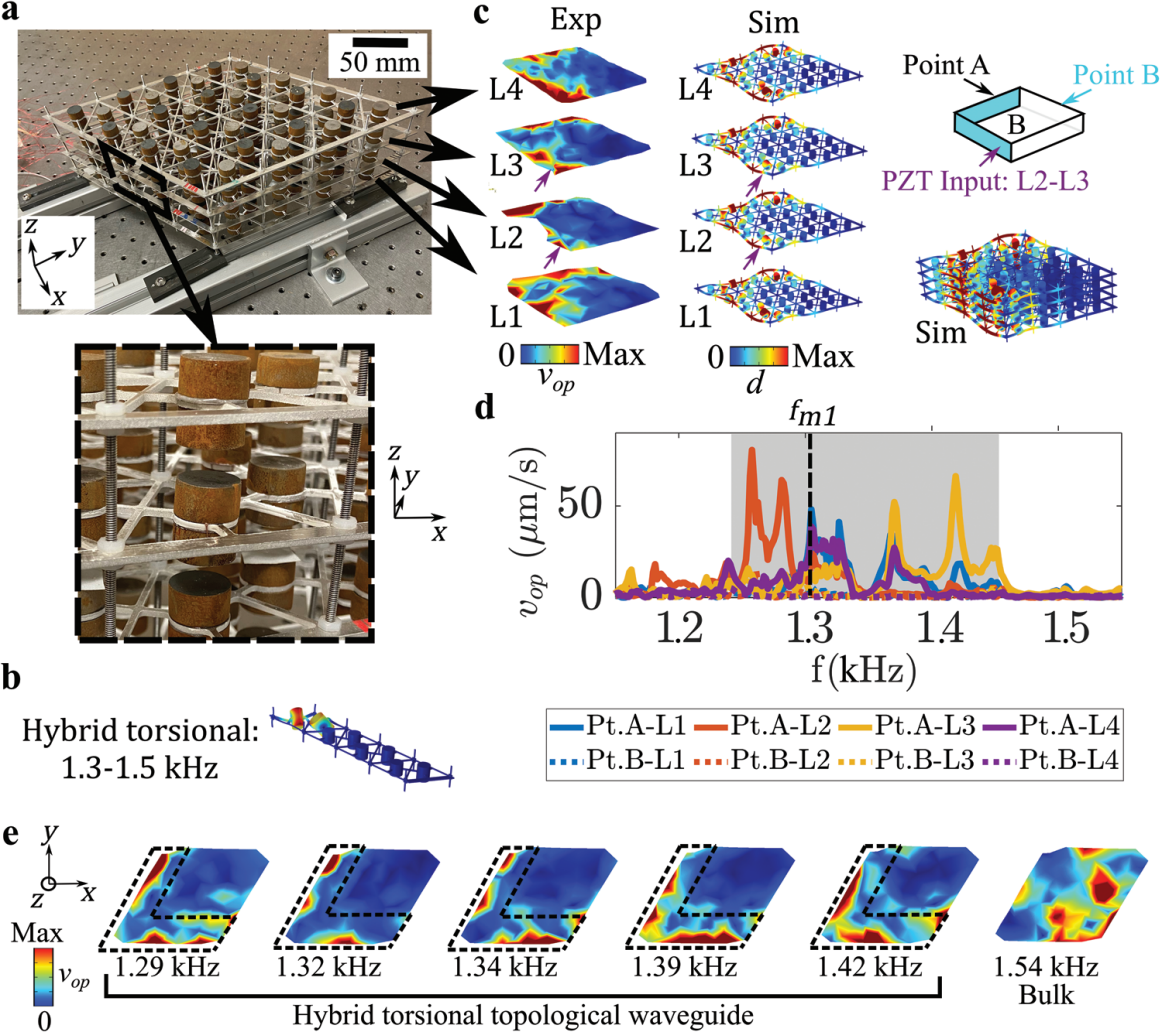
a) The experimental testbed with an inset showing detail for a unit cell. b) A topological boundary state with hybrid torsional polarization that is found in the band structure of a four‐unit supercell. c) Experimentally measured out‐of‐plane velocity field (left) and finite element simulated displacement field (right) for the 3D metastructure obtained at *f*_*m*1_ = 1.3 kHz. For clarity, both the full‐scale and layer views of the simulated displacement field are shown. The schematic of the 3D metastructure testbed is given in the top right, where the blue shading represents the V‐shaped waveguide. d) The experimentally measured out‐of‐plane velocity (*v*_*op*_) for Point A (solid lines) and Point B (dashed lines) on each of the four layers. The frequency range for effective waveguiding is marked by the gray shading. e) Experimentally measured out‐of‐plane velocity fields for L4 that illustrate the topological waveguide across a wide frequency range. A bulk response is demonstrated at 1.54 kHz.

An experimental investigation is undertaken to validate the theoretical predictions and implement the proposed 3D metamaterial in a practical setting. The experimental testbed is a 3D metastructure made up of a 4 × 4 × 4 tessellation of the Type B metamaterial unit cell (**Figure** **5**a, see Section S8, Supporting Information, for a detailed description of the fabrication and experimental testing). A supercell analysis (for a four‐unit supercell) reveals a topological boundary state with a hybrid torsional polarization that emerges in the frequency range of 1.3–1.5 kHz (Figure 5b). This boundary state is employed to design a V‐shaped waveguide in the 3D metastructure, as indicated by the blue shading in the schematic located in the top right corner of Figure 5c. A periodic chirp vibration input with a bandwidth of 0–5 kHz is provided at the middle two layers (L2 and L3) of the metastructure as indicated in Figure 5c. The excitation is provided by a piezoelectric (lead zirconate titanate, or PZT) actuator pair that provides a harmonic bending displacement in the *z* direction. A scanning laser Doppler vibrometer (SLDV, Polytec PSV‐500) is used to acquire non‐contact measurements for the out‐of‐plane velocity magnitude $v_{\mathrm{op}}=\;\left|\dot{w}\right|$, which only requires the use of a single laser. The measurements are obtained for all four layers L1–L4 of the metastructure by guiding the laser in between the gaps in the layers closest to the vibrometer head. An out‐of‐plane velocity *v*_*op*_ field taken at *f*_m1_ = 1.3 kHz illustrates the successful confinement of the dynamic response within the designated 2D waveguide and closely aligns with finite element simulations (Figure 5c, an animation of the dynamic response on L1 is found in Movie S1, Supporting Information). The frequency response is gathered for a point inside the waveguide (Point A in Figure 5c) and a point outside the waveguide (Point B in Figure 5c) on each of the four layers. The frequency response displayed in Figure 5d reveals that there is a wide frequency range (indicated by gray shading, from 1.26 to 1.47 kHz) where the out‐of‐plane velocity *v*_op_ taken at the point inside the waveguide (Point A, results represented by solid lines) is significantly higher than the velocity at the point outside the waveguide (Point B, results represented by dashed lines) for all four layers (see Section S9, Supporting Information, for additional frequency response data). Observation of multiple velocity fields for L4, which are shown in Figure 5e, confirms that the desired waveguiding behavior is occurring across the frequency range identified in Figure 5d. These results represent a clear advancement, in that this is the first time that full‐field dynamic response information illustrating a 2D topological waveguide has been experimentally acquired for vibrations in a 3D mechanical structure.

**Figure 6:**
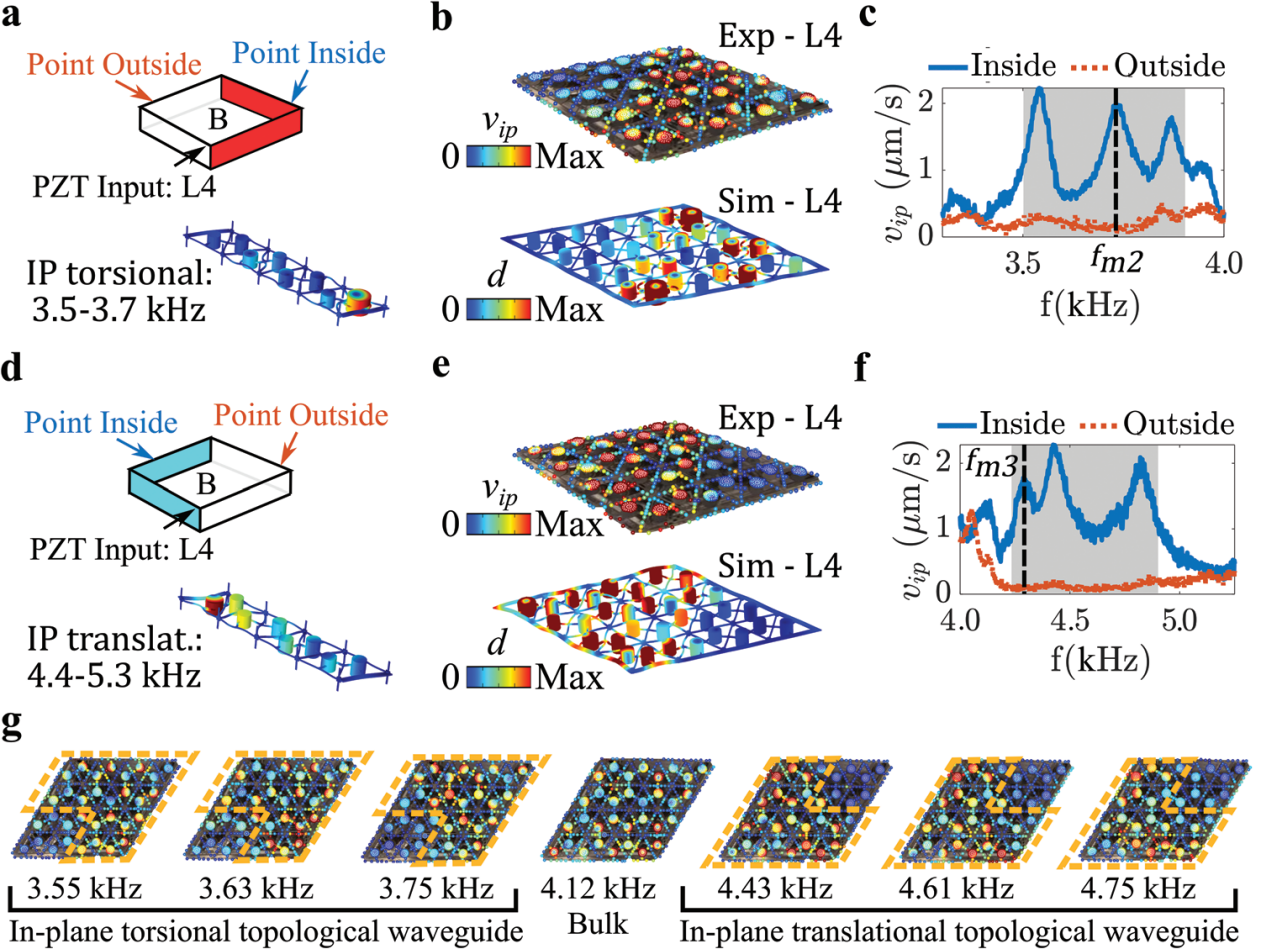
a) (top) A schematic of the 3D metastructure testbed where the red shading represents the path of a V‐shaped waveguide. (bottom) A topological boundary state with an in‐plane torsional polarization that is found in the band structure of a four‐unit supercell. b) Experimentally measured in‐plane velocity field and finite element simulated displacement field for L4 of the 3D metastructure obtained at *f*_*m*2_ = 3.7 kHz. c) The experimentally measured in‐plane velocity (*v*_*ip*_) for a Point Inside and a Point Outside the waveguide on L4. The frequency range for effective waveguiding is marked by the gray shading. d) (top) A schematic of the 3D metastructure testbed where the blue shading represents the path of a V‐shaped waveguide. (bottom) A topological boundary state with an in‐plane translational polarization that is found in the band structure of a four‐unit supercell. e) Experimentally measured in‐plane velocity field and finite element simulated displacement field for L4 of the 3D metastructure obtained at *f*_*m*3_ = 4.4 kHz. f) The experimentally measured in‐plane velocity (*v*_*ip*_) for a Point Inside and a Point Outside the waveguide on L4. The frequency range for effective waveguiding is marked by the gray shading. See Section S9 (Supporting Information) for the transmission ratio plots that accompany c) and f). g) Experimentally measured in‐plane velocity fields for L4 that illustrate the in‐plane topological waveguides across a wide frequency range. A bulk response is demonstrated at 4.12 kHz.

The in‐plane torsional and in‐plane translational topological states are also experimentally characterized in the 3D metastructure. An in‐plane torsional state (shown at the bottom of **Figure** **6**a) is used to construct a waveguide that follows the two planar boundaries identified by the red shading in Figure 6a. A piezo‐stack actuator is utilized to apply an in‐plane periodic chirp (with a bandwidth of 0–10 kHz) excitation to L4 of the structure. The in‐plane velocity components $\dot{u}$ and $\dot{v}$ are measured using a 3D SLDV (Polytec PSV QTec 3D) and the total in‐plane velocity magnitude is calculated as $v_{\mathrm{ip}}=\sqrt{|\dot{u}{|}^{2}+|\dot{v}{|}^{2}}$. The in‐plane measurements require the use of three lasers, and thus the only surface accessible for measurement is L4, the layer closest to the 3D SLDV head. The velocity field measured at *f*_*m*2_ = 3.7 kHz shows that the dynamic response follows the waveguide created by the in‐plane torsional state and matches finite element simulations (Figure 6b, animations are found that display the in‐plane torsional response in Movies S2 and S3, Supporting Information). The frequency response measured for a point inside the waveguide and a point outside the waveguide (see the schematic in Figure 6a) reveals that waveguiding behavior occurs over the frequency range of 3.5–3.8 kHz (Figure 6c), which is enclosed within the bandwidth (3.5–3.7 kHz) of the in‐plane torsional state. The same experimental approach is followed for the in‐plane translational topological state. A waveguide is constructed to follow the blue surfaces in Figure 6d and experimental measurements illustrate waveguiding at *f*_*m*3_ = 4.4 kHz (Figure 6e, animations are found that display the in‐plane translational response in Movies S4 and S5, Supporting Information). Results displayed in Figure 6f indicate that the wave field follows the prescribed path over a frequency range of 4.3–4.9 kHz, which overlaps with the bandwidth (4.4–5.3 kHz) of the in‐plane translational state reported in Figure 6d. To complement the frequency response calculations, the in‐plane velocity fields for multiple different frequencies are displayed in Figure 6g, illustrating in‐plane waveguiding behavior across the bandwidths identified in Figure 6c,f. The experimental velocity fields for the in‐plane waveguides shown in Figure 6b,e,g reveal less localized dynamic response (the majority of the response in these cases is confined within two unit cells of the boundary, as indicated by the dashed lines in Figure 6g) than the hybrid torsional waveguide reported in Figure 5c,e (the majority of the response in the hybrid torsional case is confined within one unit cell of the boundary, as indicated by the dashed lines in Figure 5e). This relatively reduced confinement is a consequence of the in‐plane topological states being less localized than the hybrid torsional state, as can be seen by comparing the mode shapes of the three topological states in Figures 5b and 6a,d. Furthermore, these differences are in agreement with the finite element simulations presented in Figure 4b, where the hybrid torsional state enables better waveguiding performance (Λ = 0.7) than the two in‐plane topological states (Λ = 0.4–0.6). Despite this comparatively lower localization, the results displayed in Figure 6 do illustrate the experimental realization of in‐plane polarized elastic wave control that follows waveguides constructed in the 3D metastructure. Altogether, the outcomes from this experimental study advance beyond the previous research on 3D topological mechanical metamaterials, which has overwhelmingly concentrated on the theoretical investigation of a single topological state,^[^ ^34^, ^35^, ^36^, ^37^, ^38^, ^39^, ^40^ ^]^ to attain experimental wave fields of topological waveguides with multiple (i.e., three: hybrid torsional, in‐plane torsional, and in‐plane translational) distinct polarizations and frequency bandwidths.

## 3 Conclusion

The research presented in this paper advances the state of the art through the creation of a 3D topological mechanical metamaterial that exploits multimodal local resonance to enable low‐frequency and multiband elastic wave manipulation. A band structure analysis reveals four topological states that emerge in distinct frequency regions that are associated with out‐of‐plane translational, hybrid torsional, in‐plane torsional, and in‐plane translational resonances. A parameter study is used to develop a deeper understanding of the connection between the frequency range for each topological state and the fundamental characteristics of the respective resonant modes (i.e., the mass and stiffness for each mode). This parametric analysis illuminates how the topological states can be attained over a broad bandwidth and at low frequencies (e.g., all below 6 kHz) by adjusting the mass and stiffness properties of the local resonators, without needing to increase the metamaterial volume. Finite element simulations of full‐scale 3D metastructures uncover how the topological states can be employed to achieve 2D planar and layer‐locked waveguides with complex frequency‐ and polarization‐dependent behaviors. To complement the theoretical findings, experiments are conducted to validate the numerical predictions and establish a benchmark for the experimental investigation of topological phenomena in 3D mechanical structures. The experimental advancements reported in this paper include the attainment of complete wave fields that unequivocally illustrate 2D topological waveguides and the measurement of multi‐polarized wave control in a 3D structure. These outcomes may inspire future experimental research on wave control and topological physics in 3D mechanical systems. In contrast to 3D topological photonic metamaterials,^[^ ^73^ ^]^ which generally operate in the microwave frequency regime, the 3D topological mechanical metamaterial described in this paper is constructed from traditional load‐bearing materials and can operate at frequencies (<10 kHz) that are relevant to structural applications. The multifaceted capabilities of the proposed 3D metamaterial could help to improve performance in engineering applications that involve low‐frequency and multiband elastic wave control, such as vibration energy harvesters and isolators.^[^ ^10^, ^11^, ^12^, ^13^, ^14^, ^51^, ^52^ ^]^ Moreover, the rich 3D, multiband, frequency‐dependent, and polarization‐dependent features of the metamaterial could be utilized to construct information‐dense phononic circuitry (i.e., elastic wave‐based, with information‐density derived from the three distinct elastic wave polarizations: two quasi‐shear and one quasi‐longitudinal) for mechanical computers, wave filters, and on‐chip devices.^[^ ^15^, ^16^, ^17^, ^18^, ^19^, ^74^ ^]^ For example, the proposed metamaterial may be used as the building block of 3D phononic circuits with multiple working channels that route waves in a layer‐dependent fashion based on the polarization and frequency of external inputs. In order to harness the full potential of the proposed metamaterial in practical applications, future endeavors may entail two primary aspects: a design optimization aimed at achieving complete topological bandgaps and enhancing wave localization in the waveguides, and an exploration of advanced manufacturing methods (e.g., multi‐material 3D printing^[^ ^75^ ^]^) to facilitate efficient production and testing of geometries featuring complex 3D waveguide paths.

## Conflict of Interest

The authors declare no conflict of interest.

## Supporting information

Supporting Information

Supplemental Movie 1

Supplemental Movie 2

Supplemental Movie 3

Supplemental Movie 4

Supplemental Movie 5

## Data Availability

The data that support the findings of this study are available from the corresponding author upon reasonable request.
