# Supplementary material for: Uncovering and Experimental Realization of Multimodal 3D Topological Metamaterials for Low‐Frequency and Multiband Elastic Wave Control
Source: Adv Sci (Weinh). 2023 Sep 4;10(30):2304793. doi: 10.1002/advs.202304793 (PMC10602582; doi:10.1002/advs.202304793)
Supplement: Supplementary file 1 — Supporting Information [file ADVS-10-2304793-s006.pdf]

ADVANCED SCIENCE
Open Access

Supporting Information

for *Adv. Sci.*, DOI 10.1002/advs.202304793

Uncovering and Experimental Realization of Multimodal 3D Topological Metamaterials for Low-Frequency and Multiband Elastic Wave Control

*Patrick Dorin, Mustafa Khan and K. W. Wang**

Supporting Information

Uncovering and experimental realization of multimodal 3D topological metamaterials for low-frequency and multiband elastic wave control

*Patrick Dorin, Mustafa Khan, Kon-Well Wang**

S1. Description of finite element simulation methods
S2. Unit cell analysis of topological bandgaps
S3. Berry curvature and valley Chern number
S4. Mode shapes illustrating multimodal resonance
S5. Design considerations for Dirac frequency separation from ligament/rod modes
S6. Supercell band structure analysis
S7. 2D multimodal topological metamaterial theory and experiments
S8. Fabrication and experimental testing
S9. Experimental frequency response
Supplemental References

Supplemental Videos (separate files):
Video S1. Animation of the experimentally measured hybrid torsional state on L1 (isometric view)
Videos S2 and S3. Animations of the experimentally measured in-plane torsional state on L4 (isometric and top views)
Videos S4 and S5. Animations of the experimentally measured in-plane translational state on L4 (isometric and top views)

S1. Description of finite element simulation methods

Simulation Methods: The COMSOL Multiphysics Solid Mechanics Module is used for all band structure and dynamic response calculations. Figure S1 contains a schematic of the unit cell used in the finite element numerical simulations. The interconnecting rods are attached to the aluminum spring ligaments via hollow cylinders that approximate the nylon coupling nuts used in the experiments (h_{nut} = 1.6 mm, $r_{nut-outer}$ = 2.4 mm, ρ_{nut} = 1140 kg□m^{-3}, ν_{nut} = 0.42, and E_{nut} = 2.41 GPa). For the purpose of this investigation, perfect adhesion is assumed between all assembled components. The finite element simulations are used to solve the governing equations for wave motion in a linear medium, represented as:[1]

$$\rho(r)\ddot{u}_i = \sum_{j=1}^{3}\left\{\frac{\partial}{\partial x_i}\left(\lambda(r)\frac{\partial u_j}{\partial x_j}\right) + \frac{\partial}{\partial x_j}\left[\mu(r)\left(\frac{\partial u_i}{\partial x_j} + \frac{\partial u_j}{\partial x_i}\right)\right]\right\} \text{for } i = 1,2,3 \ \#(\text{S1})$$

where $r = (x, y, z)$ is a position vector, $\rho(r)$ is material density, $u(r) = [u, v, w]^T$ is the displacement vector, $\mu(r)$ and $\lambda(r)$ are elastic constants, and $i, j = 1,2,3$ correspond to the coordinates x,y,z. Floquet-Bloch periodic boundary conditions are applied and a harmonic plane wave solution is used to calculate the band structure for the unit cell. A solution of the form $u(r;t) = \tilde{u}(r)e^{i(k\cdot r)}e^{i\omega t}$ is assumed, where $k = \{k_x, k_y, k_z\}$ is the Bloch wave vector, $\tilde{u}(r)$ is the Bloch displacement vector, t is time, and ω is the temporal frequency. To construct the band structure, the eigenfrequencies are calculated for k that is swept along the boundaries of the irreducible Brillouin zone.

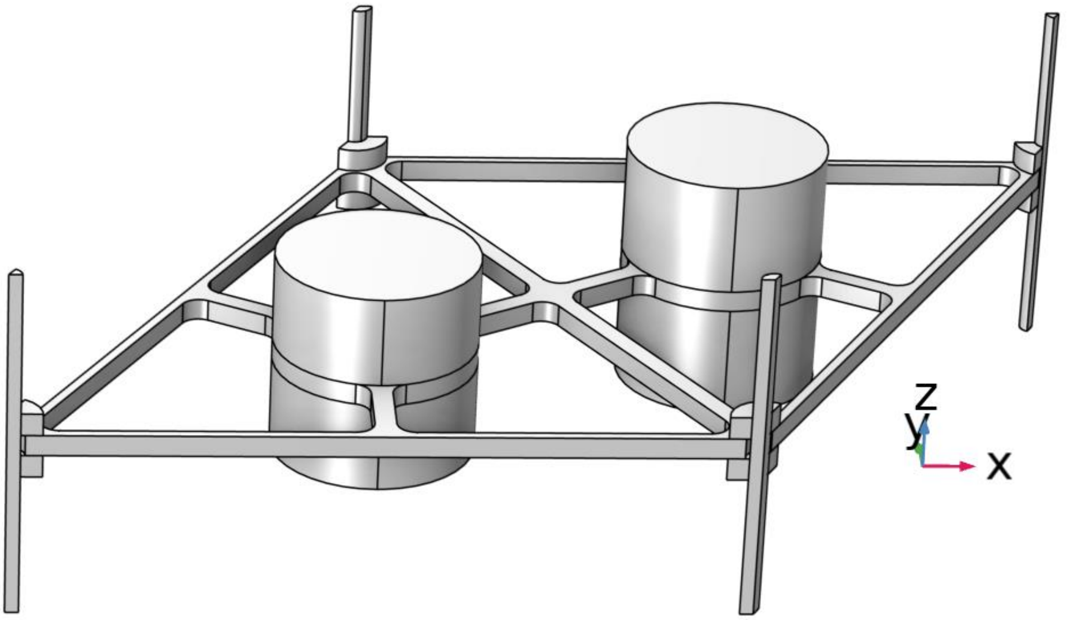

Figure S1. COMSOL model for the metamaterial unit cell used in finite element simulations.

S2: Unit cell analysis of topological bandgaps

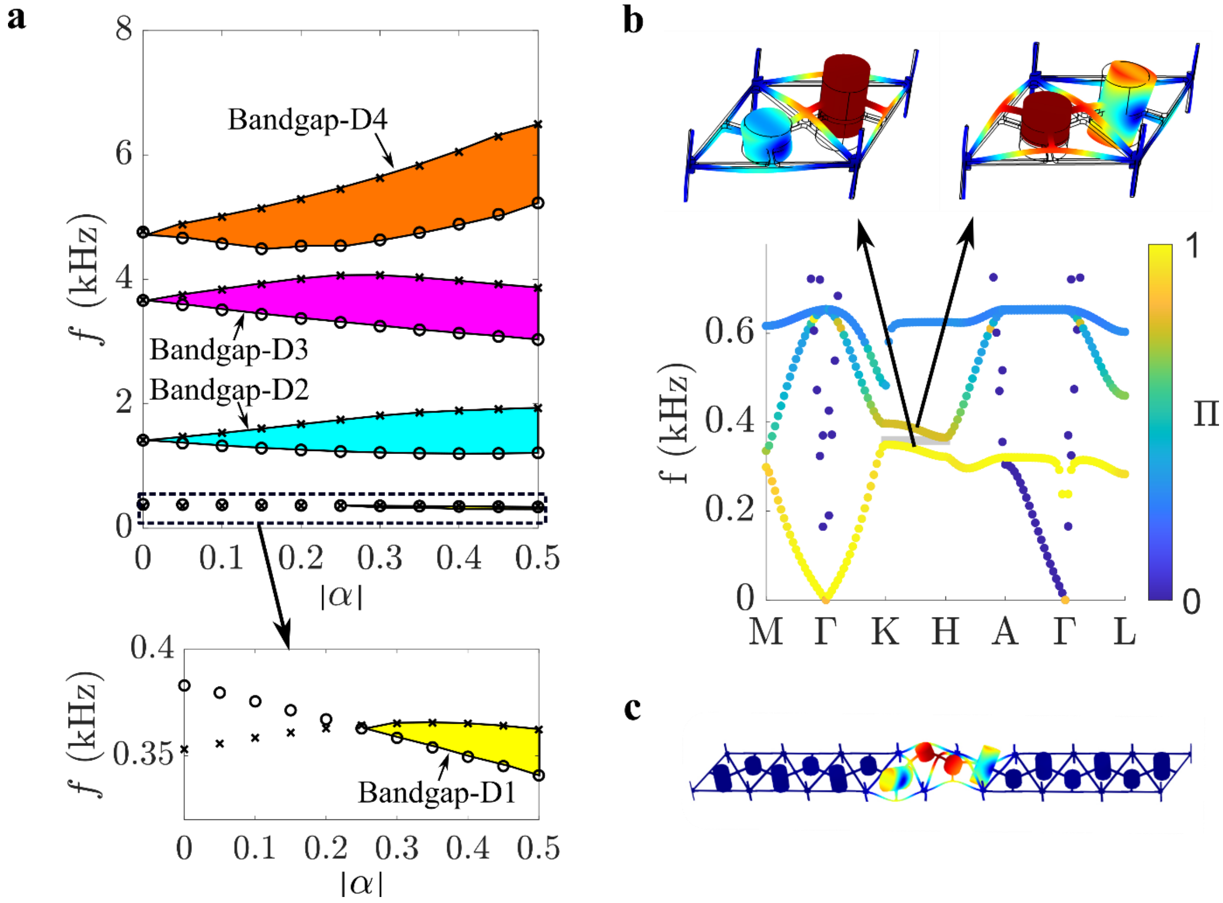

Figure S2. a) Evolution of the D1, D2, D3, and D4 partial bandgaps as a function of the mass perturbation magnitude $|\alpha|$. Open circles and crosses denote the lower and upper boundaries of the bandgaps opened from the four Dirac degeneracies. The D1, D2, D3, and D4 bandgaps are represented by the yellow, cyan, purple, and orange shaded regions, respectively. b) Band structure for the large mass perturbation ($|\alpha|$ = 0.40) case. The topological partial bandgap opened from the Dirac degeneracy D1 is marked with gray shading. The mode shapes for the bands bordering the bandgap illustrate the out-of-plane translational displacements of the resonators ($\Pi \approx 1$). c) An interface mode that emerges within the D1 topological bandgap with displacement confined at the interface between the Type A and Type B segments in an eight-unit supercell.

S3. Berry curvature and valley Chern number

The topological invariant, or the valley Chern number C_{v-p}, is calculated for each of the bands bordering the topological bandgaps that emerge from D2, D3, and D4 (Figure 1d of the main text). C_{v-p} is calculated as:[2,3]

$$A_p(k) = \langle u_p(k)|i\nabla_k|u_p(k)\rangle \#(S2)$$

$$B_p(k) = \nabla_k \times A_p(k) \#(S3)$$

$$C_{v-p} = \frac{1}{2\pi}\iint_v B_p(k)d^2k \#(S4)$$

where $A_p(k)$ is the Berry Connection, $B_p(k)$ is the Berry Curvature, $\nabla_k = \left(\frac{\partial}{\partial k_x}, \frac{\partial}{\partial k_y}\right)$ is a differential operator, $u_p(k)$ are the eigenvectors associated with the pth band (where p = 1 refers to the band delineating the low-frequency bandgap boundary and p = 2 refers to the band delineating the high-frequency bandgap boundary), and the subscript v denotes that the integral is taken around the K or H valley in reciprocal space. The eigenvectors are extracted using COMSOL with MATLAB. To ensure smoothness in the phase ambiguity over k_x-k_y, the eigenvectors are processed by a gauge transformation $|u_p(k)\rangle \rightarrow e^{i\theta^{(p)}(k)}|u(k)\rangle$, where $\theta^{(p)}(k) = \angle w^{(p)}(r_{ref}, k)$ is a reference phase calculated at an r_{ref} with non-vanishing $w^{(p)}(r_{ref}, k)$.[4,5] The eigenvectors are then normalized according to:

$$\langle u_p(k)|u_p(k)\rangle = \iiint_{V_U} u_p^*(k)u_p(k)dV \approx \sum_{N_{pts}} V_{pt}\left(u_{pt}^*u_{pt} + v_{pt}^*v_{pt} + w_{pt}^*w_{pt}\right) = 1 \#(S5)$$

where N_{pts} is the number of considered Gauss points, V_{pt} is equal to the approximate volume around each Gauss point calculated in COMSOL, and u_{pt}, v_{pt}, and w_{pt} are the x, y, and z components of the eigenvector at each Gauss point. Equation S2 is evaluated as:

$$A_p(k) = i\iiint_{V_U}\left[\left(u^*\frac{\partial u}{\partial k_x} + v^*\frac{\partial v}{\partial k_x} + w^*\frac{\partial w}{\partial k_x}\right)\hat{\imath} + \left(u^*\frac{\partial u}{\partial k_y} + v^*\frac{\partial v}{\partial k_y} + w^*\frac{\partial w}{\partial k_y}\right)\hat{\jmath}\right]dV \cong$$
$$\sum_{N_{pts}} V_{pt}\left[\left(u_{pt}^*\frac{\partial u_{pt}}{\partial k_x} + v_{pt}^*\frac{\partial v_{pt}}{\partial k_x} + w_{pt}^*\frac{\partial w_{pt}}{\partial k_x}\right)\hat{\imath} + \left(u_{pt}^*\frac{\partial u_{pt}}{\partial k_y} + v_{pt}^*\frac{\partial v_{pt}}{\partial k_y} + w_{pt}^*\frac{\partial w_{pt}}{\partial k_y}\right)\hat{\jmath}\right] = A_{k_x}\hat{\imath} + A_{k_y}\hat{\jmath}$$
$$(S6)$$

MATLAB is then used to evaluate Equation S2-S6, with the calculated C_{v-p} values listed in Table S1 and the calculated $B_p(k)$ shown in Figure S3, S4, and S5.

Table S1. Valley Chern numbers calculated for D2, D3, and D4

Valley Chern Number	D2	D3	D4
$C_{v-1}^{Type\ A}$	0.11	-0.14	0.25
$C_{v-2}^{Type\ A}$	-0.09	0.17	-0.30
$C_{v-1}^{Type\ B}$	-0.11	0.15	-0.26
$C_{v-2}^{Type\ B}$	0.09	-0.18	0.30

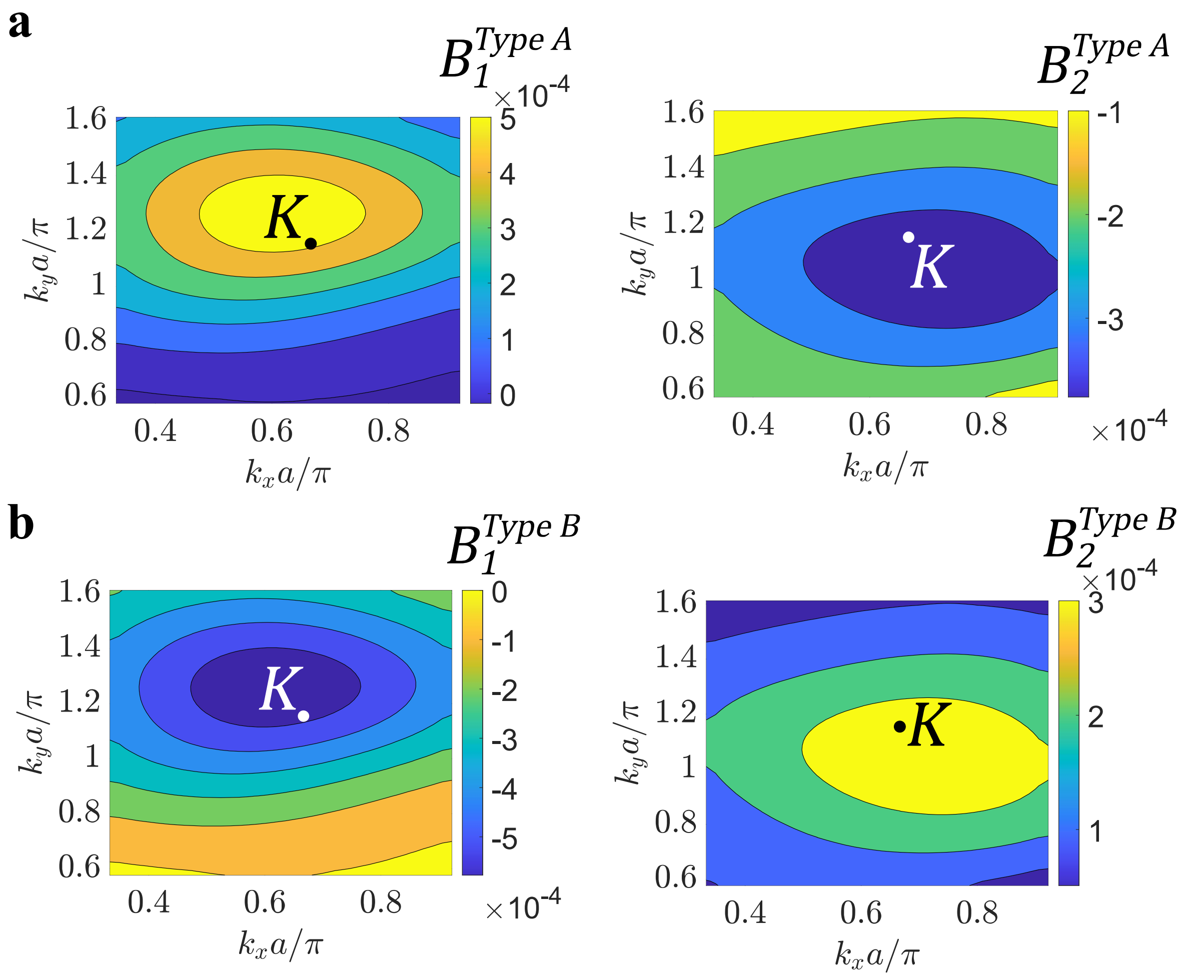

Figure S3. Berry Curvature $B_p(k)$ evaluated for the bands bordering the topological bandgap that emerges from D2. a) $B_p(k)$ for band 1 ($B_1^{Type\ A}$) and band 2 ($B_2^{Type\ A}$) of the Type A lattice. b) $B_p(k)$ for band 1 ($B_1^{Type\ B}$) and band 2 ($B_2^{Type\ B}$) of the Type B lattice. The colorbars indicate the magnitude of $B_p(k)$. The opposite $B_p(k)$ values illustrate the band inversion between Type A and Type B lattices.

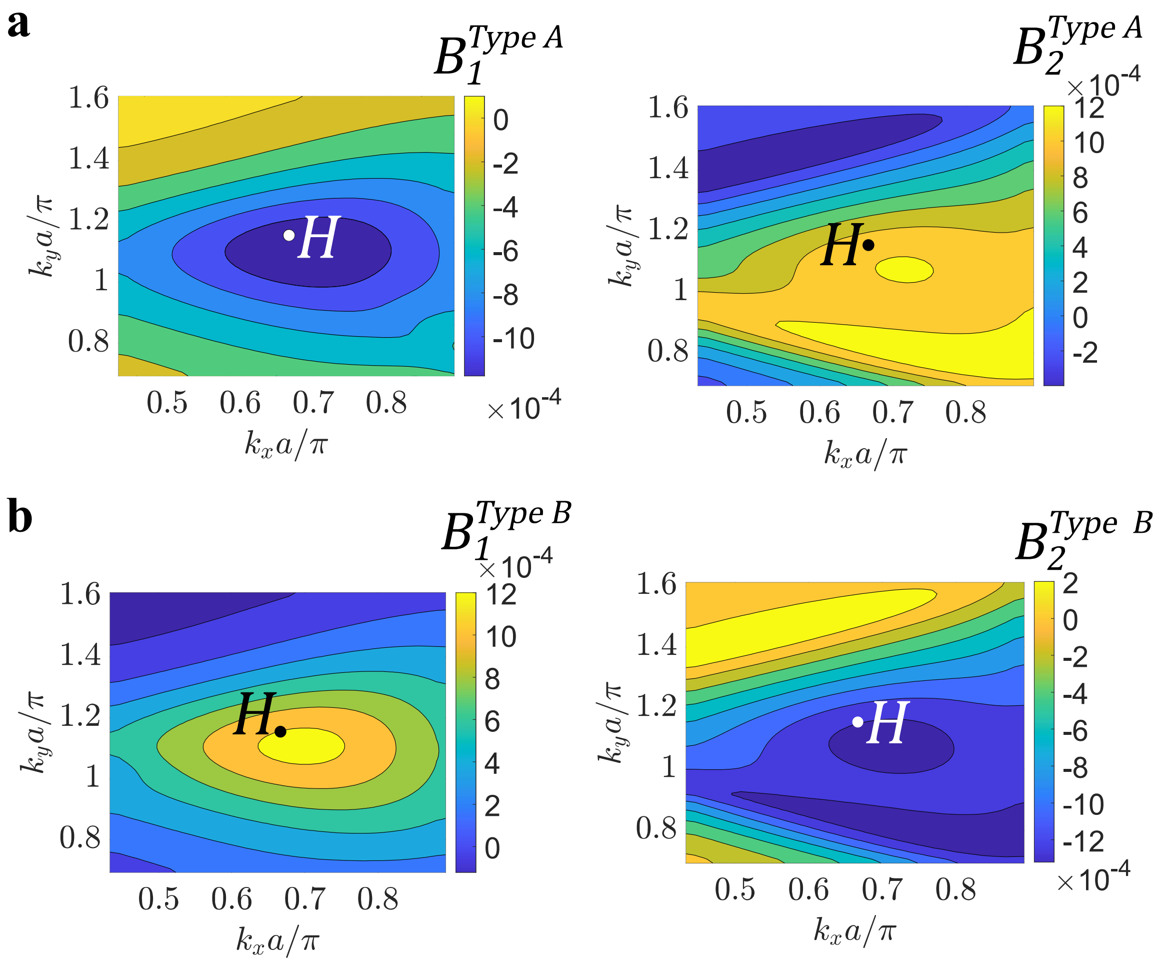

Figure S4. Berry Curvature $B_p(k)$ evaluated for the bands bordering the topological bandgap that emerges from D3. a) $B_p(k)$ for band 1 ($B_1^{Type\ A}$) and band 2 ($B_2^{Type\ A}$) of the Type A lattice. b) $B_p(k)$ for band 1 ($B_1^{Type\ B}$) and band 2 ($B_2^{Type\ B}$) of the Type B lattice. The colorbars indicate the magnitude of $B_p(k)$. The opposite $B_p(k)$ values illustrate the band inversion between Type A and Type B lattices.

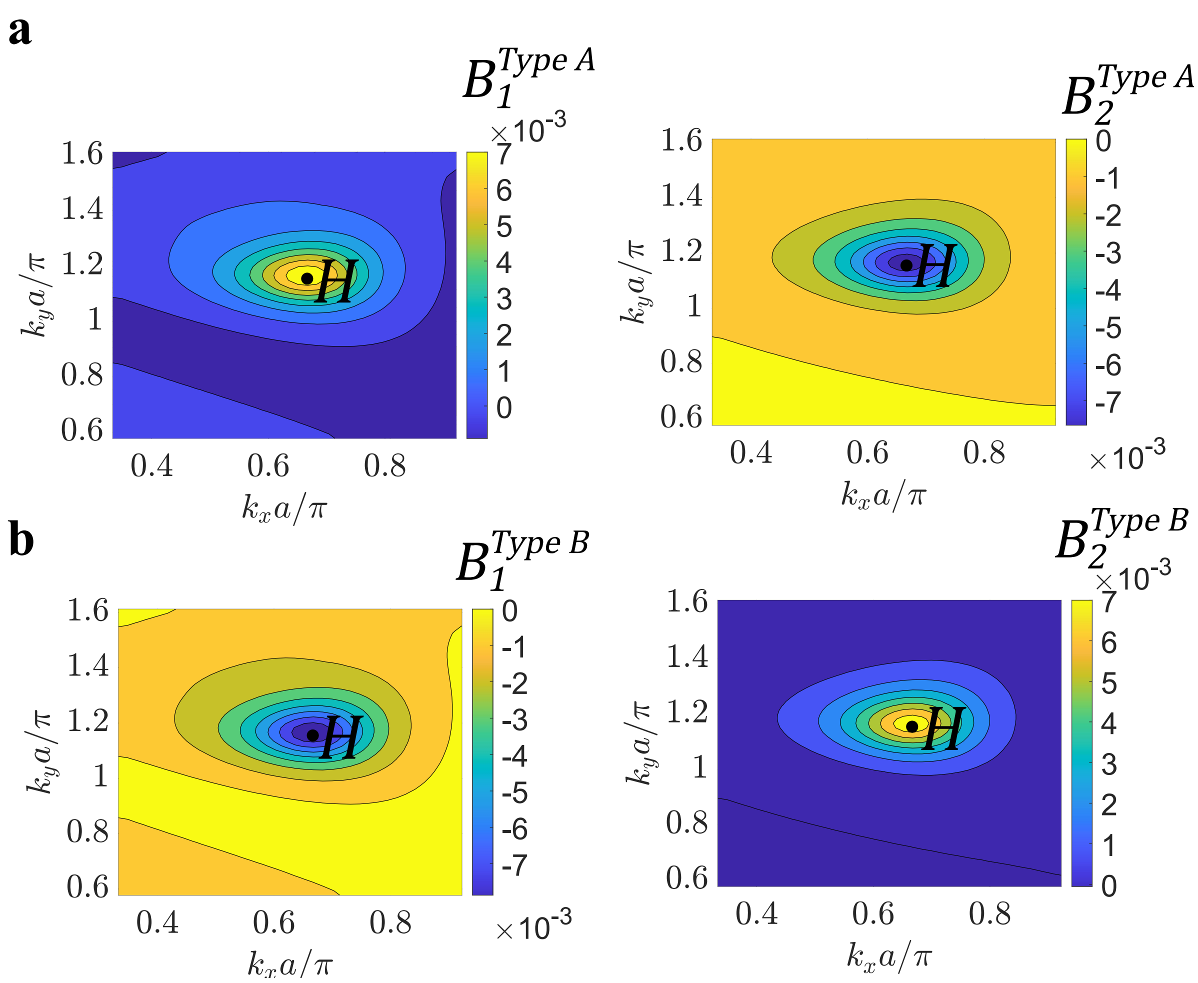

Figure S5. Berry Curvature $B_p(k)$ evaluated for the bands bordering the topological bandgap that emerges from D4. a) $B_p(k)$ for band 1 ($B_1^{Type\ A}$) and band 2 ($B_2^{Type\ A}$) of the Type A lattice. b) $B_p(k)$ for band 1 ($B_1^{Type\ B}$) and band 2 ($B_2^{Type\ B}$) of the Type B lattice. The colorbars indicate the magnitude of $B_p(k)$. The opposite $B_p(k)$ values illustrate the band inversion between Type A and Type B lattices.

S4. Mode shapes illustrating multimodal resonance

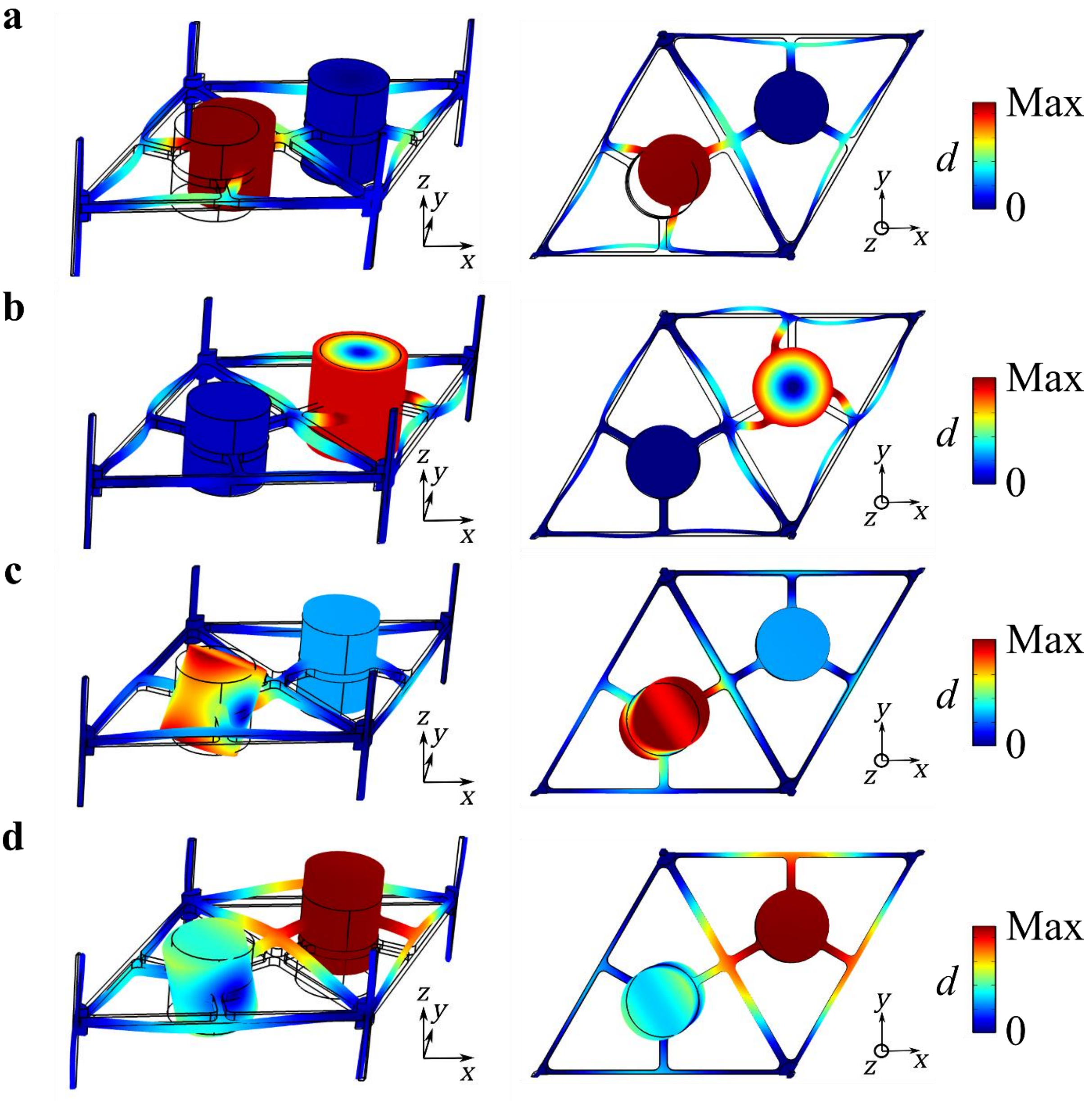

Figure S6. Selected mode shapes for the bands bordering the four topological bandgaps that emerge when $|\alpha| = 0.11$, illustrating the four primary resonant modes of the metamaterial: a) D4: in-plane (x-y) translational at 5.0 kHz (Π≈0), b) D3: in-plane (x-y) torsional at 3.5 kHz (Π≈0), c) D2: hybrid torsional at 1.5 kHz (Π=0.6), and d) D1: out-of-plane translational at 0.36 kHz (Π≈1). Both isometric and top views are provided. These mode shapes are taken for a wave vector k specified at the midpoint of the K-H line in the reciprocal unit cell.

S5. Design considerations for Dirac frequency separation from ligament/rod modes

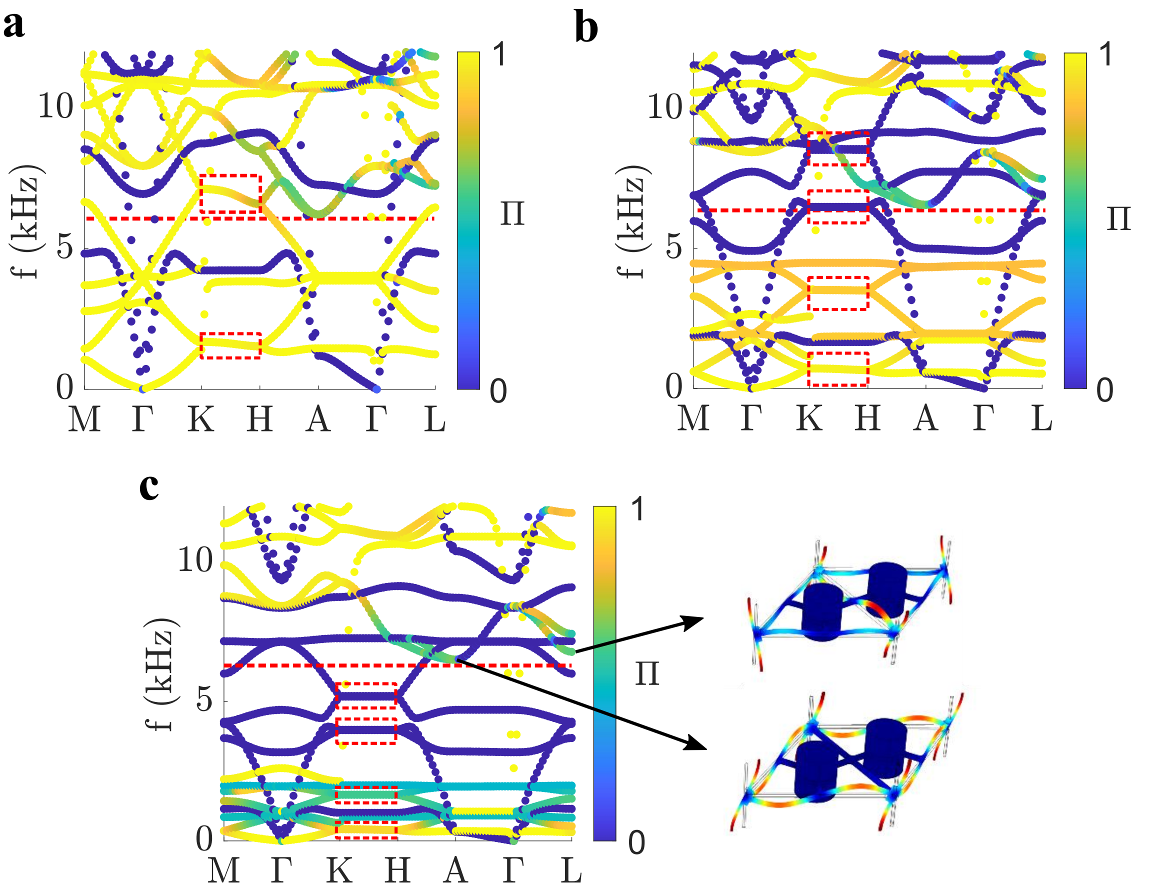

Figure S7. Band structure for the α = 0 case with a) h_m = 0 mm (no mass), b) h_m = 4 mm, and c) h_m = 12 mm. The Dirac degeneracies are enclosed in dotted red boxes. These plots illustrate how increasing the resonator mass lowers the frequency f_d of the D1, D2, D3, and D4 (f_{d-D1}, f_{d-D2}, f_{d-D3}, and f_{d-D4}) degeneracies and separates them from the ligament/rod modes that exist above 6.5 kHz. The horizontal red dashed line indicates the frequency (6.5 kHz) where the undesirable ligament/rod modes emerge that may interact with the topological bands and inhibit the successful formation of topological bandgaps and waveguides. The insets illustrate how these modes are dominated by the displacements of the aluminum ligaments and interconnecting rods. For the h_m = 0 mm (no mass) scenario in a), only D1 and D2 are visible at frequencies less than 12 kHz, and the band structure is heavily contaminated with modes that would make it difficult to open topological bandgaps. This dense band structure is a common feature of 3D elastic metamaterials, and makes the realization of low-frequency and multiband topological states in 3D structures challenging. Conversely, for a higher value of h_m = 12 mm, all four degeneracies D1-D4 are well separated from the ligament/rod modes in the frequency domain (i.e., f_{d-D1}, f_{d-D2}, f_{d-D3}, and f_{d-D4} are all well below the red dashed line) and are thus not susceptible to interactions with them.

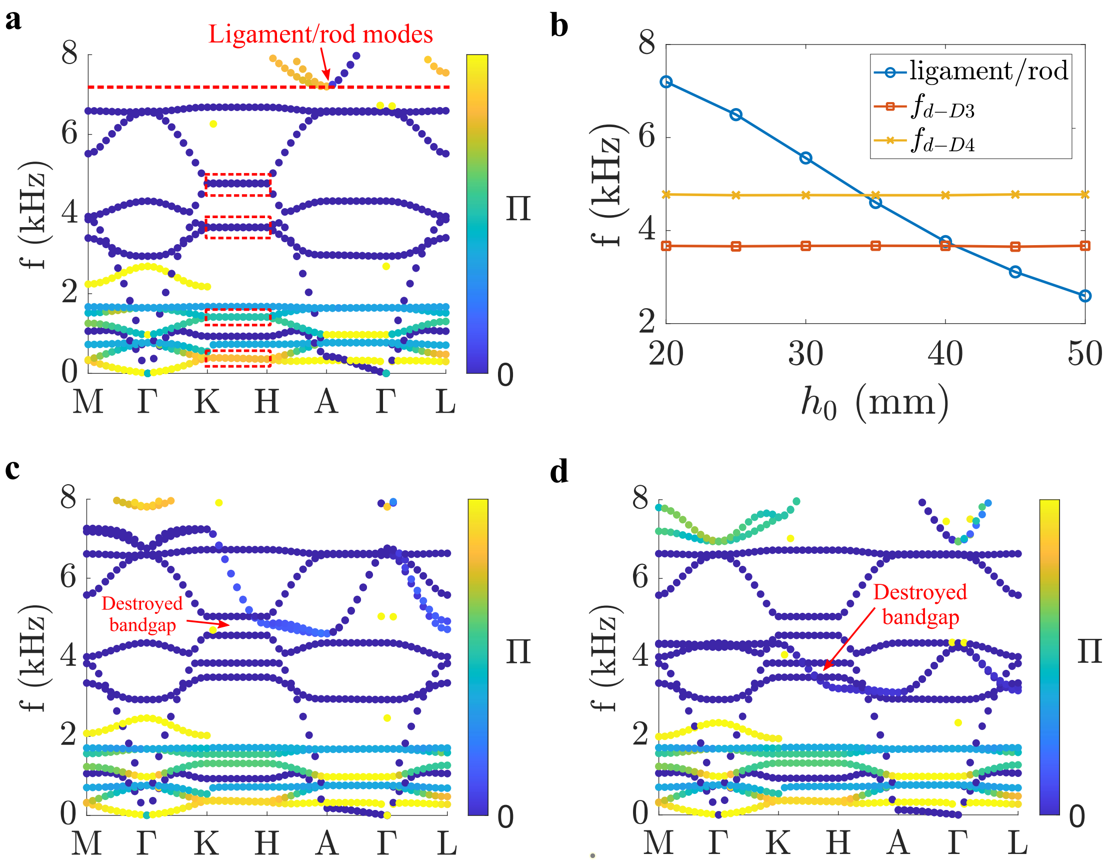

Figure S8. Analysis establishing the design criteria for the out-of-plane lattice constant h_0, which defines the height of the interconnecting rods. a) Band structure for α = 0 and h_0 = 20 mm. The Dirac degeneracies are enclosed in dotted red boxes and the horizontal red dashed line indicates the lowest frequency of the ligament/rod modes. For this case, the ligament/rod modes emerge at 7.20 kHz, well distanced from the D3 and D4 Dirac degeneracies that are located at f_{d-D3} = 3.66 kHz, and f_{d-D4} = 4.76 kHz. b) The ligament/rod mode frequencies are evaluated as a function of h_0. As h_0 is increased, the mass of the interconnecting rods increases and the lateral stiffness descreases, leading to a reduction in the ligament/rod mode frequency boundary. For $h_0 \geq 35$ mm, the ligament/rod modes overlap with f_{d-D3} and f_{d-D4}. The band structures for $|\alpha|$ = 0.11 are calculated with c) h_0 = 35 mm and d) h_0 = 45 mm. These band structures provide examples of why overlap of the topological bands with the ligament/rod modes is detrimental to the formation of topological bandgaps. The negative effects of this scenario are twofold: (i) the topological bandgaps are destroyed by the ligament/rod mode band crossing and (ii) the topological properties, as quantified by valley Chern number calculations (see Section S3), are contaminated. An out-of-plane lattice constant of h_0 = 25 mm is chosen in the main text to ensure that no undesired band crossing occurs and provide sufficent mechanical clearance for $|\alpha|$ up to a value of $|\alpha|$ = 0.50.

S6. Supercell band structure analysis

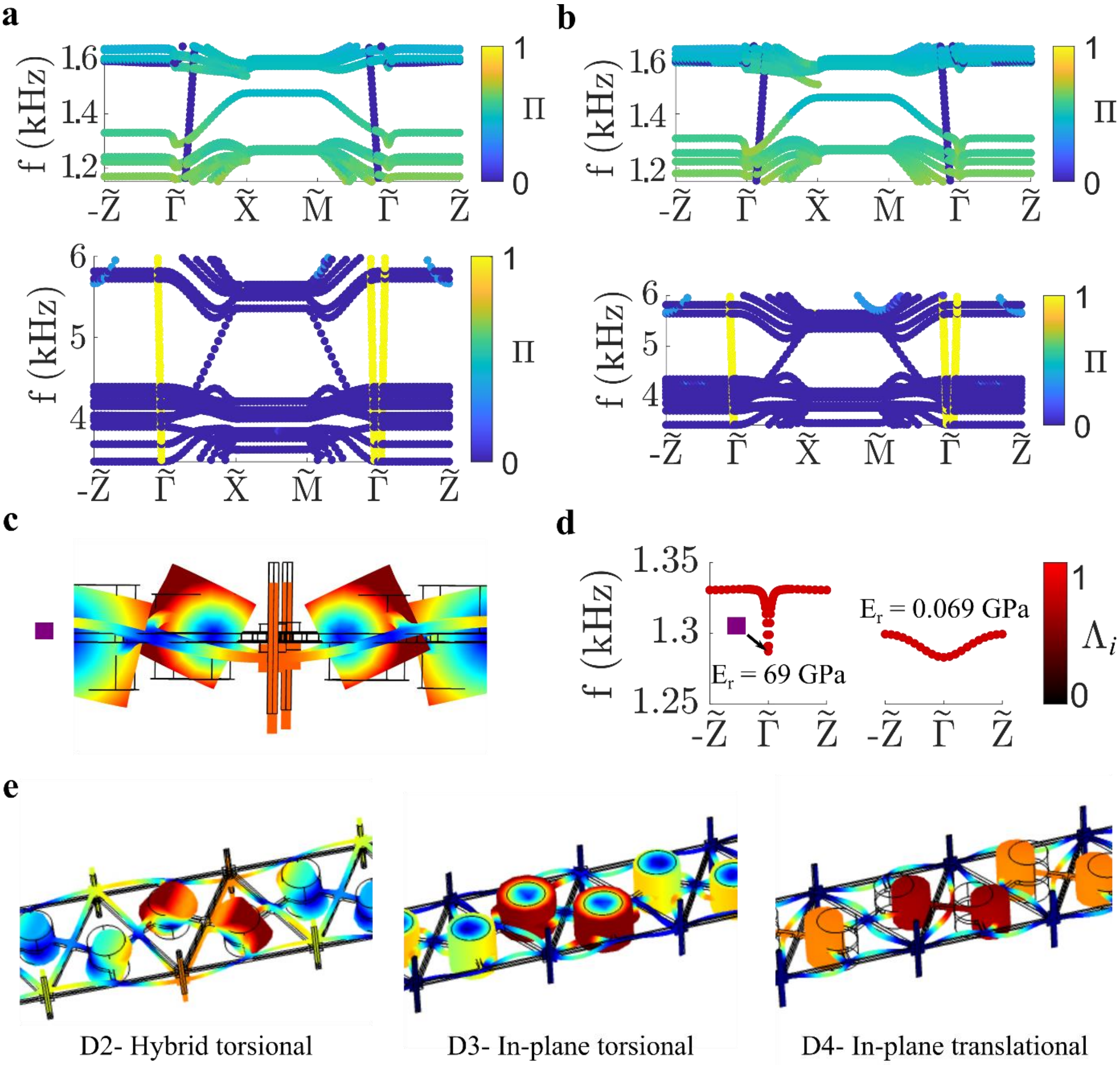

Figure S9. a) The band structure for the supercell with a Type I interface presented in Figure 3a of the main text. b) The band structure for the supercell constructed from all Type B unit cells presented in Figure 3d of the main text. In a) and b), the blue bands (Π≈0) are in-plane modes, the yellow bands (Π≈1) are out-of-plane modes, and the green bands (Π≈0.5) are modes with mixed polarization. The bulk modes that pass through the D2 (1.3 to 1.5 kHz) and D4 (4.5 to 5.1 kHz) bandgaps contain different polarizations than the respective topological states that exist in each of the bandgaps and do not visibly interact (e.g., veering or modal energy exchange) with them. These characteristics limit the possibility of unwanted mode hybridization or coupling of bulk modes with the topological states. c) A mode shape taken at Γ̃ (see the purple square in d) that illustrates the quasi-rigid body motion of the interconnecting rods. d) Demonstration of how the supercell (eight-unit supercell with Type I interface) band structure can be tailored by adjusting the elastic modulus of the

interconnecting rods E_r. For aluminum rods (E_r = 69 GPa), wave energy transmission in the z direction is limited to the long wavelength regime for a frequency range of 1.28 to 1.33 kHz. Above 1.33 kHz, the $\tilde{\Gamma}$-$\tilde{Z}$ directional bandgap leads to layer-locked wave propagation (e.g., given a 1.4 kHz excitation in Figure 4d of the main text). On the other hand, for rods made from a rubber-like material (E_r = 0.069 GPa), the frequency range of z direction transmission shrinks to 1.28 to 1.30 kHz and the portion of the band with nonzero group velocity extends beyond the long wavelength regime. e) A comparison of the supercell modes for the D2 hybrid torsional, D3 in-plane torsional, and D4 in-plane translational topological states. All mode shapes are evaluated at $\tilde{\Gamma}$. The mode shapes illustrate how the D2 hybrid torsional state transmits energy in the –z direction by coupling the hybrid torsional resonant mode with the motion of the interconnecting rods. The D3 and D4 in-plane resonances do not couple well with the interconnecting rods (which show negligible displacement in those cases) and as a result do not propagate elastic waves in the z direction, creating layer-dependent behavior.

S7. 2D multimodal topological metamaterial theory and experiments

The concept discussed in the main text is generalizable to 2D systems as well. A theoretical study (Figure S10) and experiments (Figure S11) are undertaken to show multimodal and multiband topological wave control in a 2D topological metamaterial. The 2D topological metamaterial is synthesized using the same parameters as the 3D topological metamaterial from the main text, minus the hex nuts and interconnecting rods.

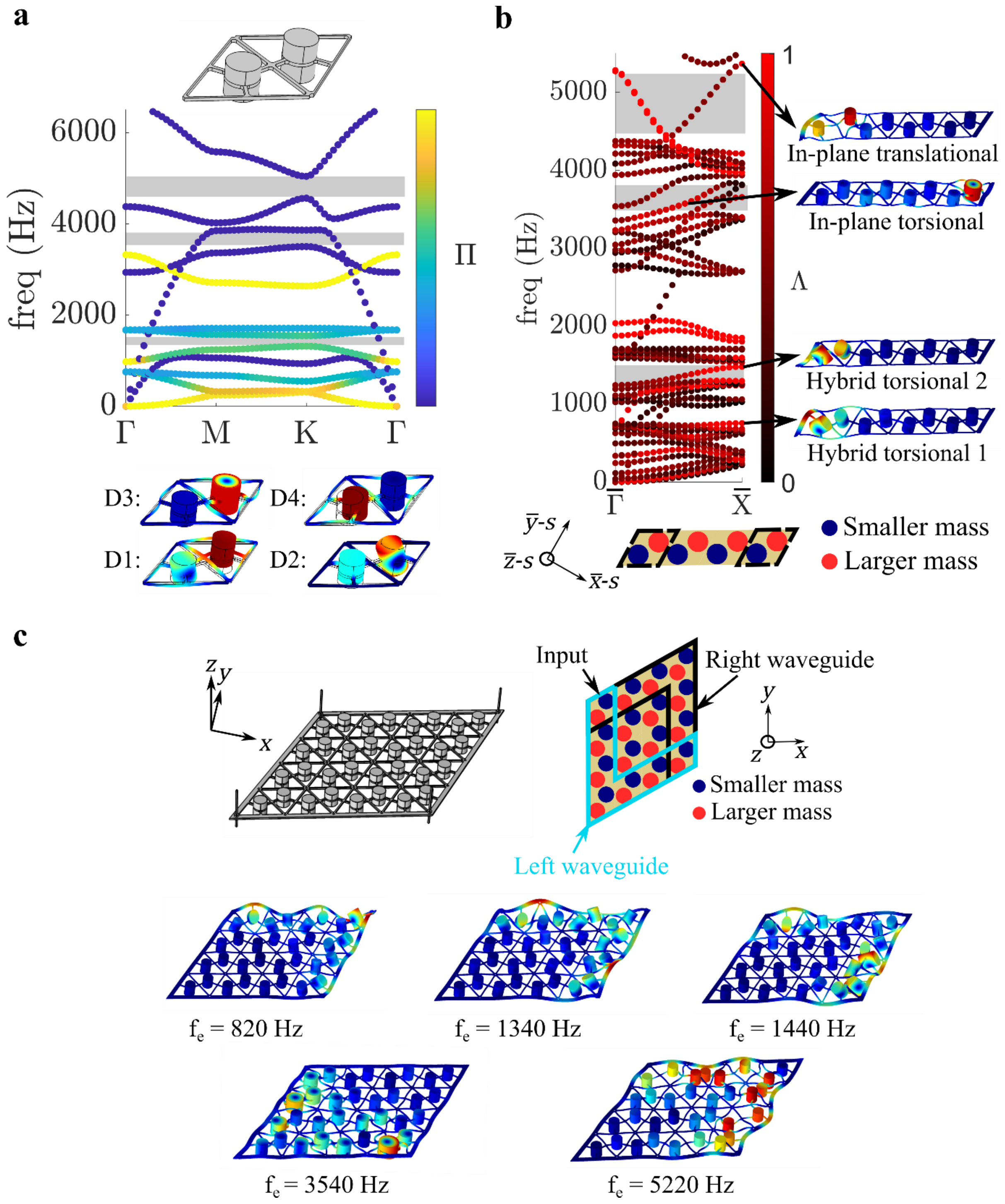

Figure S10. Theoretical investigation of the 2D topological metamaterial. a) Unit cell analysis. b) Supercell study. c) Full-scale simulations of topological wave control.

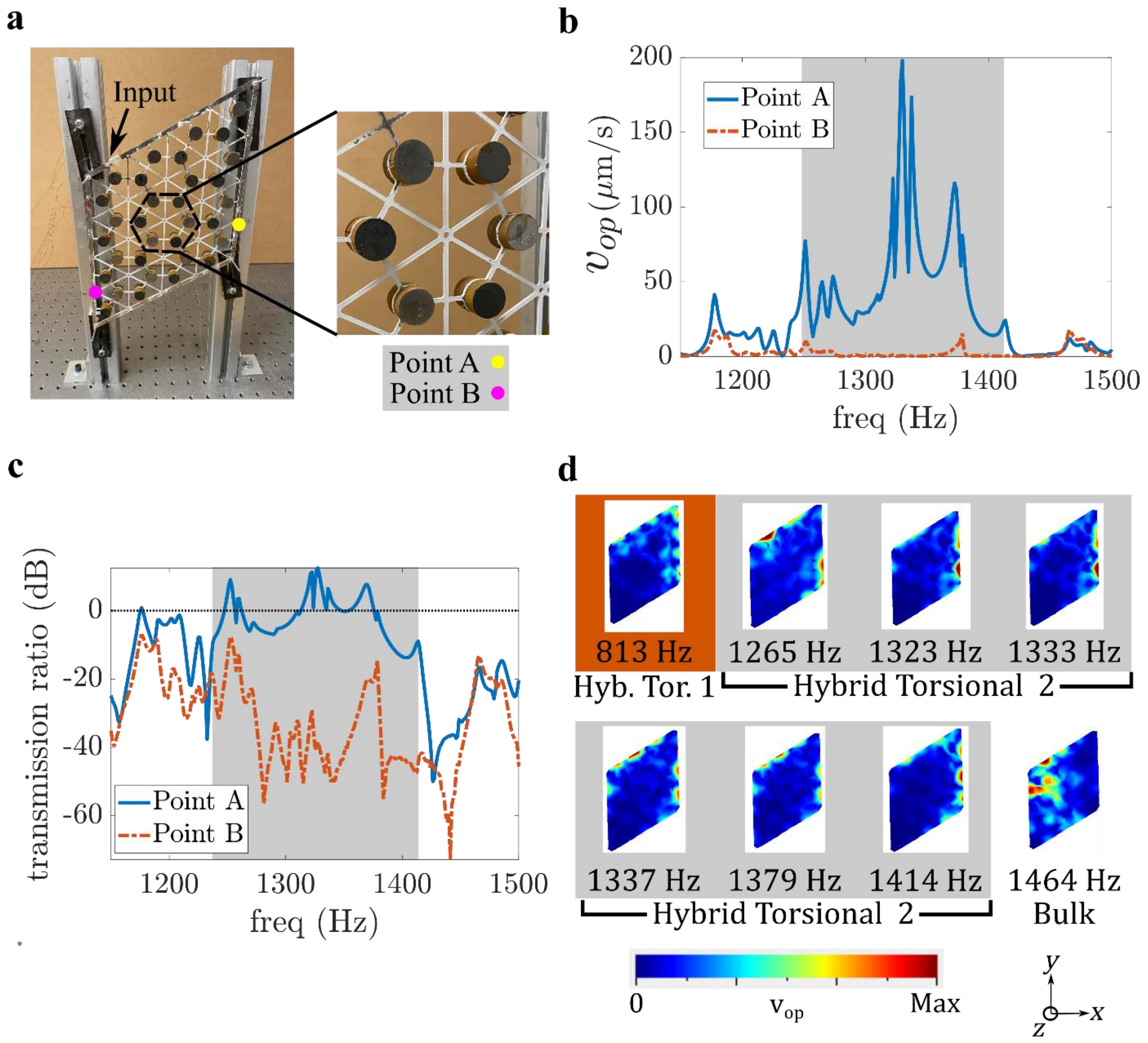

Figure S11. Experimental investigation of wave control in the 2D topological metamaterial. a) A schematic of the experimental setup. b) Experimentally measured out-of-plane velocity magnitudes (v_{op}) for the frequency range of the hybrid torsional 2 topological waveguide. c) Experimentally measured out-of-plane transmission ratio for the hybrid torsional 2 topological waveguide, where the input location is treated as the reference. d) Steady-state wave fields illustrating topological waveguiding achieved by activating the hybrid torsional 1 and hybrid torsional 2 topological states illustrated in Figure S10b. The experimental results reveal successful confinement of the dynamic response into the designated waveguide and agree with the theoretical predictions displayed in Figure S10c.

S8. Fabrication and experimental testing

Fabrication: The experimental testbed was constructed as an assembly of multiple components (Figure S12a). The hexagonal pattern was waterjet (OMAX) cut into four separate 1.5 mm thick aluminum (6061 alloy) sheets to create substrate layers with spring ligaments (i.e., to create the resonator springs). To form the resonator masses, cylinders with a diameter of 7 mm and two distinct height dimensions (7.9 mm for Mass 1 and 6.4 mm for Mass 2 in Figure S12a) were waterjet cut from low-carbon (1018 cold-finished) steel bars. The masses were glued to the aluminum substrate using steel-reinforced epoxy (Permatex® Steel Weld Epoxy). Aluminum threaded interconnecting rods (2-56 threads, 102 mm length) and nylon 6/6 hex nuts were used to connect the four aluminum layers and form the 3D metastructure. To maintain the correct spacing between layers, rectangular spacers were 3D printed using the Formlabs Form 3 photopolymer printer and used to align the layers during assembly. For testing, the four outermost (i.e., farthest from the center) interconnecting rods were fastened to a base fixture (Figure 5a of the main text).

Experimental testing: STEMiNC piezoelectric ceramic (lead zirconate titanate, PZT) disc transducers (with a radius of 5 mm and a thickness of 0.4 mm) were glued (MG Chemicals 8331-14G Silver Conductive Epoxy) to the metastructure in a bimorph configuration to provide a flexural (z direction, out-of-plane) excitation (Figure S12b). For the in-plane actuation, piezoelectric stack actuators (PICMA® P-882.11) were attached to the structure using Endevco Accelerometer Mounting Wax (Figure S12b). A schematic of the entire experimental setup is given in Figure S12c. A Polytec PSV-500 scanning laser Doppler vibrometer (SLDV) was used to acquire the out-of-plane measurements. Since only one laser was required for out-of-plane velocity ($\dot{w}$) measurements, the laser was guided through the gaps in the layers closest to the vibrometer head to reach all four layers, such that a full wave field could be acquired for the entire 3D structure. For the out-of-plane testing, a 90V (using a voltage amplifier) periodic chirp with a bandwidth of 0 to 5 kHz was sent to the PZT transducers. For the in-plane measurements, a 4V periodic chirp with a bandwidth of 0 to 10 kHz was sent as an input to the piezoelectric stack actuator and the Polytec PSV QTec 3D SLDV was used to measure all three velocity components ($\dot{u}, \dot{v}, \dot{w}$). Since three lasers were required in this case, the only layer available for measurements was the top layer, L4. All measurement data was post-processed using the Polytec Data Acquisition Center and MATLAB.

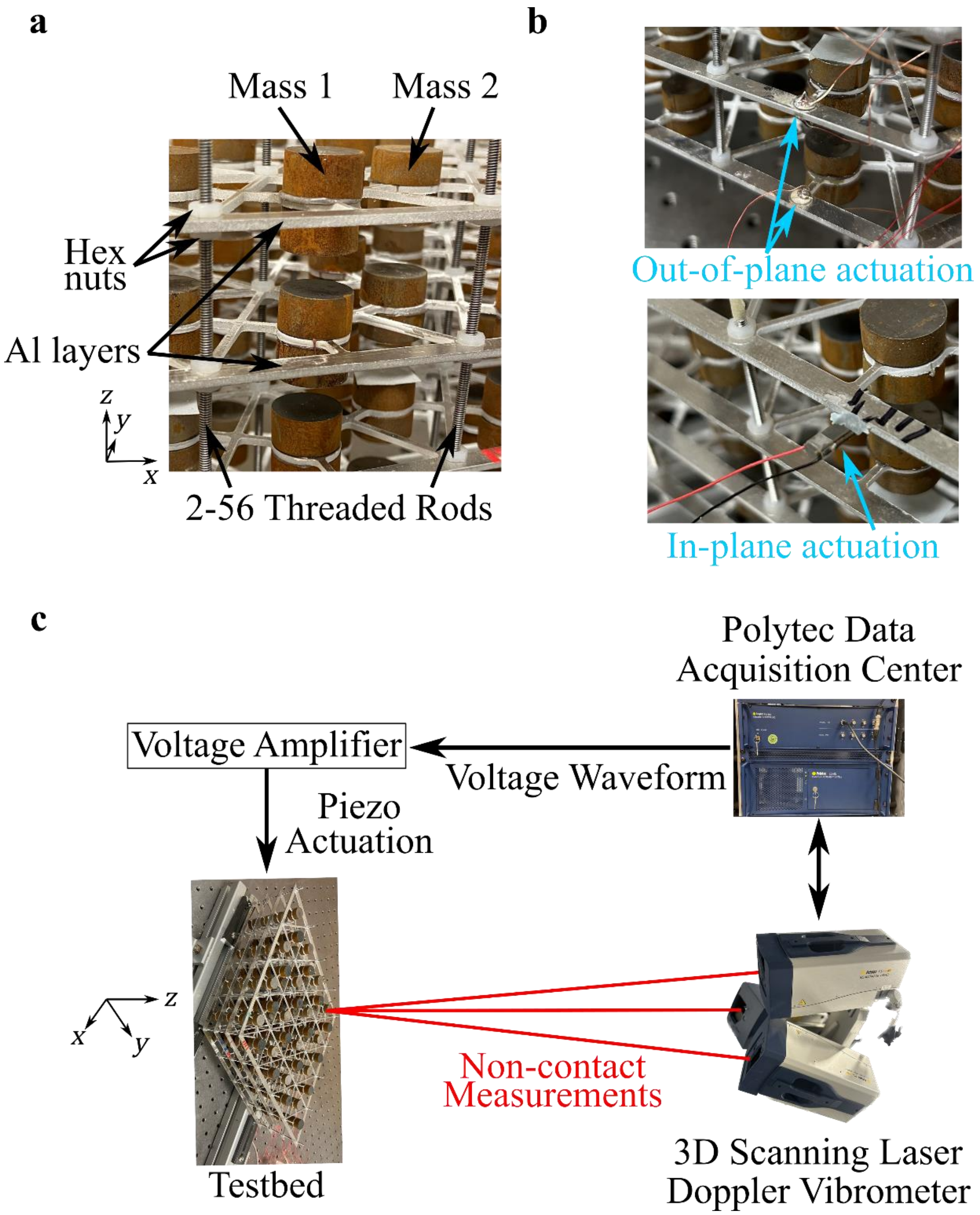

Figure S12. a) Components of the 3D metastructure assembly. b) The out-of-plane and in-plane piezo-actuators. c) A schematic of the experimental setup. In-plane measurements require the use of all three lasers, out-of-plane measurements only require the use of one laser.

S9. Experimental frequency response

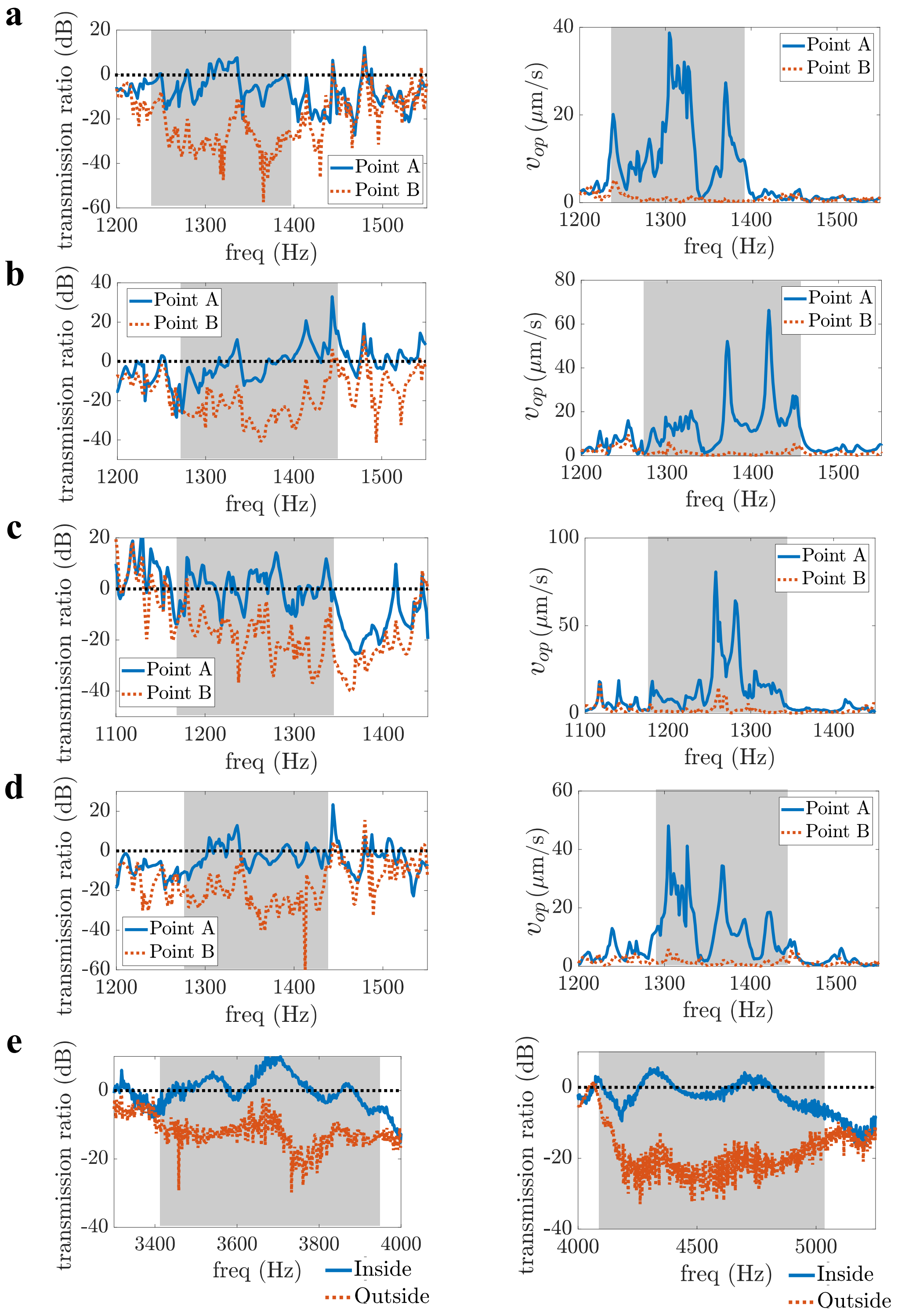

Figure S13. Experimentally measured transmission ratio (left column) and out-of-plane velocity magnitude v_{op} (right column) for a) L4, b) L3, c) L2, and d) L1 of the 3D metastructure. e) Transmission ratio for the in-plane torsional (left) and in-plane translational (right) experimental measurements. All transmission ratios are defined using the relevant PZT input location as the reference. The frequency ranges where the wave field is trapped in the designated waveguide are indicated by gray shading.

Supplemental References:

1. Graff K. Wave Motion in Elastic Solids. New York, NY, United States: Dover Publications; 1991.
2. Berry MV. Quantal phase factors accompanying adiabatic changes. Proc R Soc London A Math Phys Sci. 1984;392(1802):45–57.
3. Xiao D, Yao W, Niu Q. Valley-contrasting physics in graphene: magnetic moment and topological transport. Phys Rev Lett. 2007;99(23):236809.
4. Liu TW, Semperlotti F. Nonconventional topological band properties and gapless helical edge states in elastic phononic waveguides with Kekulé distortion. Phys Rev B. 2019;100:214110.
5. Liu TW, Semperlotti F. Synthetic Kramers Pair in Phononic Elastic Plates and Helical Edge States on a Dislocation Interface. Adv Mater. 2021;33(9):2005160.
